# Hypoxia-inducible factor-2α promotes fibrosis in non-alcoholic fatty liver disease by enhancing glutamine catabolism and inhibiting yes-associated protein phosphorylation in hepatic stellate cells

**DOI:** 10.3389/fendo.2024.1344971

**Published:** 2024-02-28

**Authors:** Ranran Yan, Hao Cai, Xiaofeng Zhou, Guodan Bao, Zhenzhong Bai, Ri-li Ge

**Affiliations:** ^1^ Qinghai-Utah Joint Key Lab for High-altitude Medicine, Medical College of Qinghai University, Xining, China; ^2^ Research Center for High Altitude Medicine, Medical College of Qinghai University, Xining, China; ^3^ Key Laboratory of High-Altitude Medicine in Qinghai University, Ministry of Education, Xining, China; ^4^ Key Laboratory for Application of High-Altitude Medicine in Qinghai Province, Xining, China; ^5^ Oncology Department, The Fifth People’s Hospital of Qinghai Provincial, Xining, China; ^6^ Affiliated Hospital of Qinghai University, Xining, China

**Keywords:** NAFLD/NASH, hepatic stellate cells-derived myofibroblasts, glutaminolysis, HIF-2α, YAP/p-YAP

## Abstract

Non-alcoholic fatty liver disease (NAFLD) has a high global prevalence and affects approximately one-third of adults, owing to high-fat dietary habits and a sedentary lifestyle. The role of hypoxia-inducible factor 2α (HIF-2α) in NAFLD progression remains unknown. This study aimed to investigate the effects of chronic hypoxia on NAFLD progression by examining the role of hypoxia-inducible factor 2α (HIF-2α) activation and that of hepatic stellate cell (HSC)-derived myofibroblasts through glutaminolysis. We hypothesised that hypoxia exacerbates NAFLD by promoting HIF-2α upregulation and inhibiting phosphorylated yes-associated protein (YAP), and that increasing YAP expression enhances HSC-derived myofibroblasts. We studied patients with NAFLD living at high altitudes, as well as animal models and cultured cells. The results revealed significant increases in HSC-derived myofibroblasts and collagen accumulation caused by HIF-2α and YAP upregulation, both in patients and in a mouse model for hypoxia and NAFLD. HIF-2α and HIF-2α-dependent YAP downregulation reduced HSC activation and myofibroblast levels in persistent chronic hypoxia. Furthermore, hypoxia-induced HIF-2α upregulation promoted YAP and inhibited YAP phosphorylation, leading to glutaminase 1 (GLS1), SLC38A1, α-SMA, and Collagen-1 overexpression. Additionally, hypoxia restored mitochondrial adenosine triphosphate production and reactive oxygen species (ROS) overproduction. Thus, chronic hypoxia-induced HIF-2α activation enhances fibrosis and NAFLD progression by restoring mitochondrial ROS production and glutaminase-1-induced glutaminolysis, which is mediated through the inhibition of YAP phosphorylation and increased YAP nuclear translocation. In summary, HIF-2α plays a pivotal role in NAFLD progression during chronic hypoxia.

## Introduction

Non-alcoholic fatty liver disease (NAFLD) and its severe form, non-alcoholic hepatitis (NASH), are chronic fatty liver diseases affecting approximately one-third of adult population worldwide. This high prevalence has been attributed to the increased popularity of high-fat dietary habits and sedentary lifestyles (1–3). NAFLD, also known as metabolic-associated fatty liver disease, is characterised by steatosis and inflammation of hepatocytes caused by metabolic factors not related to alcohol consumption (4, 5). Moreover, NAFLD can progress to NASH due to unknown reasons in the absence of effective treatment options, leading to cirrhosis and end-stage primary hepatic carcinoma (6). Identifying potential molecular mechanisms underlying NAFLD progression may contribute to the development of an effective treatment. The activation of hepatic stellate cells (HSC)–derived myofibroblasts is the major cause of NAFLD progression (7, 8). In addition, glutamine catabolism is the main source of energy for HSC-derived myofibroblasts during NASH and progression to hepatic carcinoma (9–11).

Obstructive sleep apnea-induced hypoxia is closely associated with NAFLD progression (12, 13) and cardiovascular events (14, 15). Therefore, understanding metabolic alterations associated with the hypoxic microenvironment of hepatocytes is essential to identify the molecular mechanisms underlying NAFLD progression. Activation and transfer of hypoxia-inducible factor (HIF)-2α to the nucleus are among the cellular responses to chronic hypoxia in chronic liver disease (16–18). Elevated HIF-2α levels in patients with NASH have been associated with inflammation and development of fibrosis (19). However, how HIF-2α affects glutamine catabolism in HSCs (known as glutaminolysis) during NAFLD progression remains unclear.

Yes-associated protein (YAP) is a key component in the Hippo signalling pathway, regulating gene expression by binding to the transcriptional co-activator TAZ and playing an important role in myofibroblasts derived from HSCs (20–23). Glutamine enters HSCs through the SLC38A1 also called Sodium Coupled Neutral Amino Acid Transporters 1 (SNAT1) and is metabolised by glutaminase 1 (GLS-1) to provide energy to myofibroblasts (24, 25). YAP promotes glutamine metabolism and activates quiescent HSCs during NAFLD progression (10, 16). Additionally, glutamine metabolism has been shown to be enhanced by blockage of the phosphorylated YAP pathway, and this was dependent on HIF-2α (26, 27). Since a role for HIF-2α has been described in chronic hypoxia associated to NASH (18, 19, 28), we hypothesised that HIF-2α may promote YAP activation, increasing energy demand and accelerating fibrosis progression. This study aimed to investigate the potential role of HIF-2α in the metabolic reprogramming of HSCs during NASH pathogenesis. The study includes data from patients with NASH, from a mouse model for the disease, and from HSCs in culture.

## Materials and methods

### Clinical data and histology analysis

Health examination results from a cohort of male patients with NAFLD (n=73) living at an altitude range of 2300–3500 m in Qinghai (China) were collected for the study (**Table 1**). The inclusion criteria were as follows: no previous history of alcoholism, a diagnosis of steatosis using ultrasound, and absence of other liver diseases. The participants were in the early stages of NAFLD, ranging from F0 to F3, and had been originally diagnosed via ultrasound as part of a routine health examination at the Affiliated Hospital of Qinghai University from 2019 to 2022. In addition, liver tissue sections from twelve patients with NASH and fibrosis (stages F2–F3) who underwent liver biopsy for liver cancer screening were collected for Haematoxylin and Eosin (H&E), immunohistochemical, and immunofluorescence staining (**Supplementary Table 1**), and plasma for LC-MS. To identify patients with hypoxemia, we divided patients with NAFLD into two groups based on haemoglobin concentrations: the hypoxemia (>210 g/L) and the normoxia (120–180 g/L) group, according to the Qinghai Criteria used to diagnose chronic mountain sickness (CMS), which includes haemoglobin concentrations above 21g/dL in men or 19g/dL in women accompanied by the specialised clinical signs (29). This study was approved by the Ethics Committee of the Medical College of Qinghai University, China (No. 2022-06).

**Table 1:** Demographic characteristics of the NAFLD patients.

Variables	Hemoglobin		P Value
	120<Hb<180g/L	Hb≥210g/L	
n	35	38	-
Ages (years)	46.26 (40.00 - 54.00)	44.26 (32.00 - 56.00)	0.05
Erythrocyte (×10^12^/L)	4.72 (4.15 - 5.18)	6.16 (5.61 - 6.58)^*^	<0.0001
Leukocyte (×10^9^/L)	5.81 (4.74 - 6.42)	6.42 (5.10 - 7.42)^*^	<0.0001
Platelet (×10^9^/L)	217.20 (173.00 - 251.00)	173.74 (137.50 - 211.75)^*^	0.04
ALT (U/L)	25.89 (15.00 - 29.00)	51.56 (24.50 - 61.00)^*^	<0.0001
Cholesterol	5.06 (4.28 - 6.01)	4.75 (3.77 - 5.49)^*^	0.09
Triglyceride	4.72 (0.85-7.43)	4.35 (1.38 - 5.50)^*^	<0.0001
Fasting Glucose	7.40 (5.00 - 8.75)	6.66 (4.53 - 6.58)^*^	<0.0001

*p < 0.05 vs hemoglobin <180 g/L group; Hb, Hemoglobin.

For immunohistochemistry (IHC) and immunofluorescent (IFC) staining, liver tissues were fixed in formalin, embedded in paraffin, sliced, de-waxed, and treated with EDTA (pH 9.0) (Servicebio G1203, Wuhan, China) to retrieve antigens. They were then incubated in an aqueous solution containing 3% hydrogen peroxide for 10 minutes to block the endogenous peroxidase. A 3% BSA (Servicebio GC305010, Wuhan, China) solution was used to blocked for 10 minutes, and after washing, they were incubated overnight at 4°C in a wet box in the presence of the GlS1 primary antibody (Proteintech Group 81486-1-RR, Wuhan, China). A hydrogen peroxide (HRP)-labelled secondary antibody (Servicebio GB23303, Wuhan, China) was used to incubate the tissue at room temperature for 50 minutes. Freshly prepared diaminobenzidine (DAB, Servicebio G1212, Wuhan, China) colorimetric solution was then added dropwise, colour development (brownish yellow) was controlled using a microscope, and the slices were rinsed with tap water to terminate the colorimetric process. The sections were re-stained with H&E for approximately three minutes to enable visualisation of the cell nuclei, dehydrated and sealed.

To determine whether HIF-1α and HIF-2α co-localise with the myofibroblasts of HSC marker α-SMA, co-staining was performed using the following primary antibodies: HIF-1α (GB114936, Servicebio, Wuhan, China), HIF-2α (GB11864, Servicebio, Wuhan, China), and α-SMA (GB111364, Servicebio, Wuhan, China), as well as HRP-labelled goat anti-rabbit IgG (GB23303, Servicebio, Wuhan, China), and Alexa Fluor 488-labelled goat anti-rabbit IgG (GB25303, Servicebio, Wuhan, China) as secondary antibodies. Details on antibodies and concentrations used are shown in **Supplementary Table 2**. The cell nuclei were stained with 4’,6-diamidino-2-phenylindole (DAPI, G1012, Servicebio, Wuhan, China). After quenching the spontaneous fluorescence of the tissue, and antifluorescence quenching sealing agent was used for sealing. Images were captured using an inverted laser confocal microscope (ZEISS LSM880, Jena, Germany).

### Liquid chromatography–mass analysis

Amino acid concentrations from collected plasma samples were measured using a SHIMADZU-LC30 ultra-high performance liquid chromatography system (UHPLC) (Nexera X2 LC-30AD, Japan) and ACQUITY UPLC HSS T3 (2.1X Perform chromatographic separation on a 100 mm, 1.8 µ m (Waters, USA) column. Each sample was detected in positive (+) and negative (-) ion modes by electric spray ionization (ESI). The raw data was aligned, retention time corrected, and peak area extracted using the MSDIAL software (MSDIAL ver.4.9, USA). Amino acid structure identification was performed using precise mass number matching (mass tolerance <10 ppm) and secondary spectrum matching (mass tolerance <0.01 Da), with the search performed in public databases such as Human Metabolites Data Base as well as self-built metabolite standard libraries (BP-DB) for providing us with LC-MS/MS analysis. For the extracted data, ion peaks with missing values greater than 50% within the group were not included in the subsequent statistical analysis. The total peak area of the positive and negative ion data were normalised separately, the positive and negative ion peaks were integrated, and the Python software (Python 3.8.10, Holland) was used for pattern recognition. The data was preprocessed using unit variance (UV) scaling and then subjected to data analysis.

### Generation of the mouse models and tissue sampling

Male C57BL/6J mice aged eight weeks were purchased from the Experimental Animal Centre of Xi’an Jiaotong University. The mice were randomly divided into four groups. The control group was fed with an standard chow diet. the NAFLD model group was fed with a high-fat diet (Research diet, D12492, US) and high-sugar water (23.1 g/L D-fructose, f0127, and 18.9 g/L d-glucose, G8270, Sigma-Aldrich, St. Louis, MO, USA). the NASH (C-NASH) model group was fed with a high-fat diet with sucrose and, and received intraperitoneal injections (0.2 mL/kg, two times/week, continuously for three weeks) of carbon tetrachloride (CCl_4_, 289116-100 mL, Sigma-Aldrich) dissolved in olive oil (O815210-500 mL, Sigma-Aldrich). the M-NASH group was fed with a methionine/choline-deficient (MCD, A02082002Bi, Ready Bite, China) diet during eight weeks (30, 31). We raised the control mice in a specific-pathogen-free animal lab, for the hypoxic group. Hypoxia (60% oxygen partial pressure) was elicited by housing the mice for 28 days in a hypobaric chamber simulating an altitude of 4500 m, DYC - 300, Guizhou Feng Lei Oxygen Chamber Co., Ltd., Guizhou, China). The experiment was reviewed and approved by the Medical College of Qinghai University, IACCU (No. 2022-06).

The mice were weighed, and the amount of food intake was recorded weekly. Blood glucose levels were measured using a blood glucose meter (Bayer, USA). Glucose tolerance tests were conducted. Each mouse was fasted for 12 h before receiving an intraperitoneal injection of 10% glucose solution (glucose; Sigma-Aldrich), with blood glucose concentrations measured immediately and after 15, 30, 60 and 120 min. The experimental animals were finally weighed, sacrificed, and the liver tissue was harvested. The tissue was weighed, and the liver index was calculated by dividing the liver weight (g) by the corresponding body weight (g).

The samples underwent protein and gene expression quantifications and were stored at a -80 °C ultra-low temperature refrigerator for subsequent testing. Whole blood centrifugation was used to separate serum samples, which were then stored at -80°C for future use. Serum alanine transaminase (ALT,C009-2-1), aspartate transaminase (AST, C010-2-1), triglyceride (TG, A110-1-1), and total cholesterol (T-CHO, A111-1-1) levels, including liver tissue TG, T-CHO levels, and Adenosine Triphosphate (ATP, A095-1-1) concentrations, were measured using biochemistry kits (Nanjing Jiancheng Bioengineering Institute, Nanjing, China).

### Histopathological analysis of liver tissue

Liver tissue samples were fixed in 4% paraformaldehyde, dehydrated, embedded, and sectioned for routine staining according to the instructions for H&E and Sirius red staining provided by the manufacturer (Solarbio, Beijing, China). Sirius red stain features three colours for tissue staining: acid fuchsin stains collagen fibres in red, aniline blue stains nuclei in blue, and phosphomolybdic acid stains muscle and cytoplasm in green. Sirius red staining was used to quantify the degree of fibrosis in liver tissues by measuring the area of collagen fibres in the tissue section, using Image J software, and quantifying the blue-stained areas in the selected regions. H&E staining was observed under an optic microscope (Olympus, Japan). We evaluated the degree of pathological changes using the NAFLD activity score (NAS). Sections subjected to Sirius red staining were observed under a microscope, and the percentage of blue-stained fibrosis was determined. For transmission electron microscopy (TEM), the harvested liver tissues were fixed in a 3% glutaraldehyde solution and sent to the Lilai Biomedicine Experiment Centre (Chengdu, China) for imaging.

### Flow cytometry

The fresh liver tissue was cut into small pieces, incubated with collagenase IV, and processed to obtain a single-cell suspension (32). Following the operating procedures for detecting the membrane-permeable JC-1 dye (C25H27Cl4N4.I) with an excitation wavelength of 514 nm, emission light of 529 nm, FITC/SSC (fluorescein isothiocyanate/side scatter) channel, and a reactive oxygen species (ROS) assay kit using the fluorescent probe 2’-7’-Dichlorodihydrofluorescein diacetate (DCFH-DA) was used to measure mitochondrial ROS production. DCFH-DA can be hydrolysed by intracellular esterases to generate DCFH without exiting the cell, allowing the probe to penetrate the membrane easily. Intracellular ROS can then oxidise non-fluorescent DCFH to generate fluorescent DCF. Therefore, the amount of DCF fluorescence was measured to estimate intracellular ROS levels. This was performed using an excitation wavelength of 300 nm, an emission light wavelength of 610 nm, and the phycoerythrin channel in a flow cytometer. A mitochondrial permeability transition pore (mPTP) assay kit was used to detect and assess the opening of the pore by the fluorescence of the mitochondrial membrane-penetrating probe calcein AM, with an excitation wavelength of 494 nm, an emission light wavelength of 517 nm, and the fluorescein isothiocyanate (FITC) or side scatter (SSC) gating channel. All three kits were obtained from Beyotime Biotechnology (Shanghai, China), and the experiments were performed using a CytoFLEX flow cytometer (Beckman, California, USA). Signal parameters were set according to the manufacturer’s instructions. The results were analysed and quantified using the CytoFLEX 2.1 software (Beckman, California, USA).

### RNA extraction and quantitative analysis

TRIZOL reagent (Tiangen, Biotech, Beijing, China) was added to liver tissues and cells to extract total RNA. The RNA was Reverse-transcribed into cDNA (Tiangen, China). **Table 2** shows the primers designed and synthesised to quantify the expression levels of candidate genes (Sangon, Shanghai, China). SYBR Green from Sagan Corporation (Shanghai, China) was used for real-time polymerase chain reaction (RT-PCR) in an ABI Q5 equipment (Applied Biosystems, Foster City, CA). All experiments were performed in triplicate for quantitative PCR (qPCR) analysis.

**Table 2:** Primers sequences and species.

Genes	Species	prime	Primer Sequences (5’→3’)
GLS1	mouse	Forward	GTCCTGAGGCAGTTCGGAATA CACG
		Reverse	AGGAGGAGACCAACACATCATGC
SLC38A1	mouse	Forward	GAGCACAGGCGACATTCTCATCC
		Reverse	CATGGCGGCACAGGTGGAAC
actin	mouse	Forward	GTGACGTTGACATCCGTAAAGA
		Reverse	GCCGGACTCATCGTACTCC
GLS1	human	Forward	GTCACGATCTTGTTTCTCTGTG
		Reverse	GTCCAAAGAGCAGTGCTTCAT CCATG
SLC38A1	human	Forward	GCTTTGGTTAAAGAGCGGGC
		Reverse	CTGAGGGTCA-CGAATCGGAG
actin	human	Forward	CATCTGCTGGAAGGTGGACA
		Reverse	CGACAGGATGCAGAAGGAGA
Col1A	human	Forward	AAAGATGGACTCAACGGTCTC
		Reverse	CATCGTGAGCCTTCTCTTGAG
α-SMA	human	Forward	TCGTGCTGGACTCTGGAGATGG
		Reverse	CCACGCTCAGTCAGGATCTTCATG

### Cell lines and culture

Considering the ability of the LX-2 human HSC line to retain key features of hepatic stellate cytokine signalling and fibrogenesis, LX-2 cells (33) were purchased (Procell Life Sciences, China) and cultured in Dulbecco’s modified eagle medium with 10% foetal bovine serum (GIBCO, US) containing 1% penicillin and 1% streptomycin (GIBCO). DMEM media with and without glutamine was purchased from Procell Life Sciences. Before stimulation treatment, a cells were seeded at a density of 2×10^5^/mL on a six-well plate, and cultivate in serum-free medium for 8 hours, and hypoxic treatment consisted of 200 μM palmitic acid (PA, P0500, Sigma-Aldrich) in 1% O_2_ for 72 h. Cell culture was conducted in CO_2_ incubator (Heracell, Thermo, US) at 37°C, 5% of CO_2_, and 21% of O_2;_ and in tri-gas CO_2_ incubators (Memmert, Shanghai, China) at 37°C, 5% of CO_2_, and 1% of O_2_.

### Wounding healing assay

Cells were seeded into 6-well plates (5 × 10^5^cells/mL) using serum-free medium. After 24 h, pipette tips were used to scratch the cell monolayer. The wound was washed to remove non-adherent cells, and culture medium without glutamine (PM150213, Procell Life Sciences, China) was added for 24 h. Cell migration into the scratched area was observed and quantified using an optic microscope. The percentage of wound closure was determined according to the following formula: wound closure rate = [wound area (0h) – wound area (24 h)]/wound area (0 h).

### Small interfering RNA transfection

To inhibit HIF-2α expression under hypoxic conditions, small-interfering RNAs (siRNAs) corresponding to the oxygen-dependent domains (ODDs) of HIF-2α were synthesised using Shanghai Genechem Gene Technology Co., Ltd (Shanghai, China). HIF-2α siRNA or scrambled siRNA was cloned into the GV493 packaging plasmids containing the luciferase gene fragment to generate a GV493-hU6-MCS-Ubiquitin-firefly_Luciferase-IRES-puromycin construct. Lentivirus containing HIF-2α siRNA was used to infect LX-2 cells seeded at a density of 1×10^5^ cells/mL in a 24-well plate with a multiplicity of infection (MOI) of 40 using the Hitrans G virus infection enhancing kit (Genechem, China) at a concentration of 5×10^7^ Tu/mL for 16 h. The selected Transfection efficiency was confirmed by detecting green fluorescent protein fluorescence signal. The protein levels of HIF-2α were down regulated by 67% which show in **Supplementary Materials**. Moreover, YAP was synthesised using OBiO technology company (Shanghai, China). YAP short hairpin RNA (shRNA) or scrambled shRNA was cloned into the pSLenti-U6 packaging plasmid with the luciferase gene fragment to generate the pSLenti-U6-shRNA-CMV-mCherry-F2A-Puro-WPRE construct. Lentivirus containing YAP shRNA was used to infect LX-2 cells seeded at a density of 1×10^5^ cells/mL in a 24-well plate with a multiplicity of infection (MOI) of 40 using the HitransG virus infection enhancing kit (Genechem) at a concentration of 5×10^8^ Tu/mL for 16 h. The cells were incubated in a medium containing 0.4 μg/mL puromycin for 72 h. The efficiency of the silencing was determined using qRT-PCR and western blot analyses. The protein levels of YAP1 were down regulated by 71% which show in **Supplementary Materials**.

### Western blot analysis

Extracted proteins from tissues and cells using Radio Immunoprecipitation Assay lysis buffer (RIPA, R0010) from Solarbio Life Sciences (Beijing, China). The BCA reagent kit from Thermo Scientific (23225, Waltham, USA) was used to detect protein concentration. Protein lysates were loaded on a 10% tris-glycine gel and transferred to a 0.22 μm polyvinylidene difluoride membrane. The primary antibodies (**Supplementary Table 3**) used to detect protein bands in the membranes included HIF-1α, YAP1, phosphorylated (p)-YAP(S127), Collagen type I alpha 1 (Col1A), mitochondrial oxide phosphorylation complex cocktails (OXPHOS), HIF-2α, α-smooth muscle actin (α-SMA), tubulin from Cell Signaling Technology (Danvers, USA) and β-actin from Sigma-Aldrich (St. Louis, US). The anti-rabbit immunoglobin G HRP-linked antibody and anti-mouse immunoglobin G horseradish from Cell Signaling Technology (Danvers, USA). The chemiluminescence method from Millipore Corporation was used to display the protein bands of western blot. The protein bands were visualised using an Amersham Imager 600 (GE, USA) and were quantified using densitometry analysis (Image J x64 software, NIH).

### Immunofluorescence

Immunofluorescence was used to identify the cellular distribution and expression levels of HIF-2α, YAP1, p-YAP (Ser 127), and Col1A in LX-2 cells. Phosphate-buffered saline (PBS)-washed cell plates were fixed in ice-cold 95% methanol and 0.2% Triton X-100. They were then blocked with a 3% BSA solution and incubated with antibodies against HIF-2α, α-SMA, YAP1 (Alexa Fluor 647 Rabbit monoclonal to active YAP1, excitation wavelength of 647 nm, emission light of 652 nm; Abcam, Cambridge, UK), p-YAP(S127), and Col1A at 4°C for 20 h. Afterwards, the cells were incubated with fluorescent secondary antibody (excitation wavelength of 488 nm, emission light of 495 nm; Abcam) in the dark for 2 h. The cell nuclei were stained with DAPI, and the plates were sealed with an anti-fluorescence quenching sealing agent. After washing with PBS, the sections were visualised and imaged using a laser confocal microscope (ZEISS LSM880, Germany). Laser confocal microscopy was used to detect the intensity in each fluorescence channel, and the results were analysed using the ZEN 2.5 software (Zeiss).

### Statistical analysis

Data were analysed using IBM SPSS Statistics 25 and GraphPad Prism 8.0 software. Partial least squares-discriminant analysis calculated models were applied to identify different metabolites from the raw LC-MS data between two groups: NASH patients with hypoxemia (Hemoglobin >210g/L) and without hypoxemia (120g/L < Hemoglobin < 180g/L). The significant discriminations of relative levels of amino acids were selected by an iterative backward selection strategy using Variable Importance in Projection (VIP) calculations. The significant discriminations of metabolites were used as values of VIP > 1, as an accepted threshold. Experimental data were obtained from multiple replicates. Inter-group data were analysed using one-way analysis of variance (ANOVA). Quantitative and normally distributed data were expressed as Mean ± Standard deviation, and inter-group data were analysed using one-way ANOVA. Variables with a skewed distribution were represented by the median and quartile, expressed as Mean (P_25_, P_75_), and the rank sum test was used for analyses involving multiple groups. Statistical significance was set at p < 0.05.

## Results

### Patients with NAFLD and hypoxaemia have increased ALT associated with augmented glutamine catabolism

**Figure 1:**
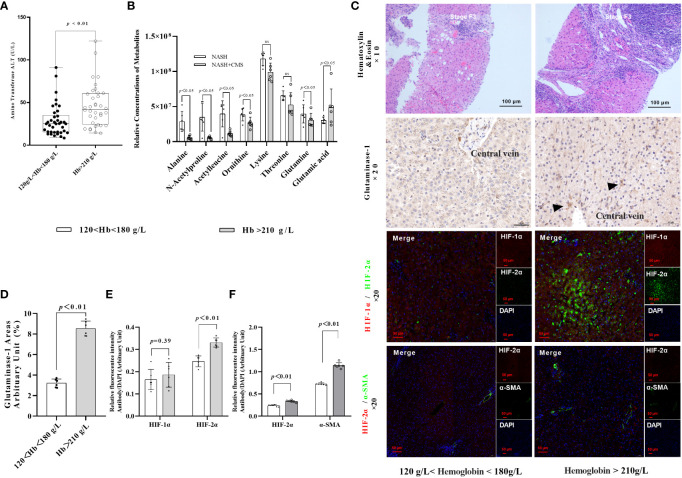
HIF-2α exacerbates fibrosis in patients with NASH by increasing GLS1 expression during chronic hypoxia. **(A)** Serum ALT levels were higher in male patients with NASH and hypoxemia (haemoglobin >180 g/L) compared to those with normal haemoglobin levels (120–180 g/L) (p < 0.01). **(B)** Relative concentration of amino acids in plasma (n=6). **(C)** H&E staining shows increased fibrosis in NASH patients with hypoxaemia. Immunohistochemistry staining shows increased GLS1 expression in liver tissues of patients with NASH and hypoxaemia. Immunofluorescence staining shows a non-significant increase in the expression of HIF-1α and a significant increase in HIF-2α expression and its co-localisation with α-SMA in liver sections from patients with NASH and hypoxemia. (n = 6; magnification, 200×). **(D)** GLS1 staining was significantly increased in patients with NASH and hypoxemia compared to those in the control group (n = 6). **(E)** HIF-2α protein levels were significantly increased in patients with NASH and hypoxemia compared to those in the control group (n = 6). **(F)** HIF-2α and α-SMA protein levels were significantly increased in patients with NASH and hypoxemia compared to those in the control group (n = 6). Data are presented as means±SD of independent experiments. H&E, Haematoxylin and eosin staining; NASH, non-alcoholic hepatitis; GLS1, Glutaminase 1; HIF-1α, hypoxia-inducible factor-1α; α-SMA, α-smooth muscle actin.

Following our previous finding of increased hepatic HIF-2α in patients with NASH living at high altitudes, we hypothesised that HIF-2α may play an additional role in NAFLD and NASH progression. We first investigated the associations between hypoxaemia and ALT in a cohort of patients with NAFLD living in high altitudes (n=73). The general characteristics of patients with NAFLD are shown in **Table 1**. Moreover, we found that male patients with NASH and hypoxaemia (haemoglobin >210 g/L) had significantly increased serum levels of ALT compared to those without hypoxaemia (**Figure 1A**). Thereafter, we found that the expression levels of glutaminase-1 were significantly higher in patients with NASH and hypoxaemia (haemoglobin >210g/L) compared to those without hypoxaemia. The glutamine levels were significantly decreased by 25%, but those of glutamate were increased by 30% in the plasma of patients with hypoxaemia (**Figure 1B**). Immunohistochemistry for glutaminase-1 on liver sections of patients with NASH confirmed by histology showed that staining was located around the central vein areas in Zone 3, which is the area of relative hypoxia in the hepatic lobules (**Figures 1C, D**; **Supplementary Figure 1**). Similarly, increased intensity for HIF-2α but not HIF-1α (**Figures 1C, E**), staining were observed in the area, accompanied by increased α-SMA staining in the hypoxaemia and NASH group (**Figures 1C, F**).

### Hypoxia exacerbated hepatic fibrosis in mice with NAFLD/NASH associated with upregulated HIF-2α and YAP-induced glutamine catabolism

**Figure 2:**
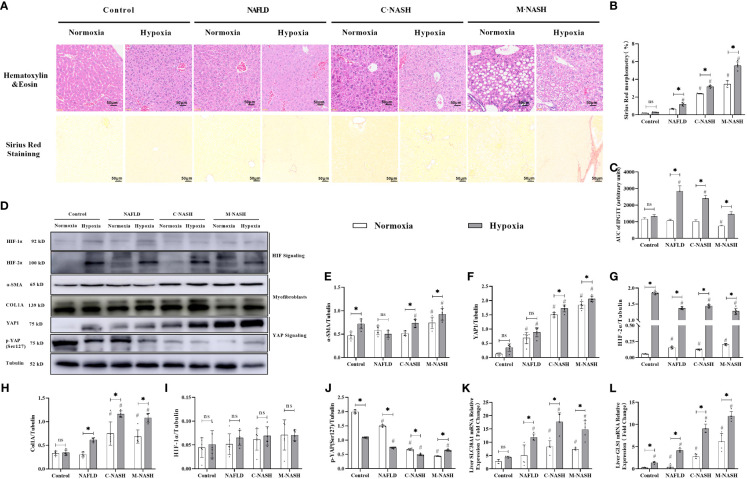
Chronic hypoxia exacerbates fibrosis in mice with NAFLD and NASH, and is associated with HIF-2α and YAP1 overexpression. **(A)** H&E and Sirius red staining of liver tissue from mouse models for NAFLD and NASH show increased fibrosis in response to hypoxia (n = 5; magnification, 200×). **(B)** Sirius red staining quantification confirmed increased fibrosis in a mouse model for NASH in response to hypoxia (n = 5). **(C)** Mice subjected to hypoxia showed impaired glucose tolerance according to the results of the glucose tolerance test. **(D)** Western blot analysis showed increased expression of α-SMA, YAP1, HIF-2α, and Col1A in mouse models subjected to hypoxia. **(E–I)** Quantification of western blot results confirmed increased expression of α-SMA, YAP1, HIF-2α, and Col1A, but not HIF-1α, in mouse models subjected to hypoxia (n = 5). **(J)** Quantification of p-YAP(S127) expression showed decreased phosphorylation of YAP in mouse models in response to hypoxia (n = 5). **(K, L)** Quantification of mRNA expression levels showed increased expression of SLC38A1 and GLS1 following hypoxia (n = 5). Bars represent mean ± SD of n=5 mice/group. *: p < 0.05 between the two groups under the same conditions, ns: p≥0.05 between the two groups under the same conditions, ^#^p < 0.05 vs Control group. NAFLD, Non-alcoholic fatty liver disease; NASH, Nonalcoholic steatohepatitis; YAP1, Yes-associated protein 1; H&E, Haematoxylin and eosin; HIF-2α, Hypoxia-inducible factor-2α; Col1A, Collagen type I alpha 1; α-SMA, α-smooth muscle actin; p-YAP(S127), Phosphorylated yes-associated protein.

Hematoxylin-eosin staining and Sirius Red staining showed increased fibrosis in the mouse models for NAFLD and NASH (**Figures 2A, B**). Moreover, severe fibrosis was observed in mice with NAFLD, C-NASH, and M-NASH subjected to hypoxia (**Figure 2B**; **Supplementary Figure 2**). Furthermore, all three groups of experimental animals showed increased NAS values (**Table 3**). No significant differences were found in food intake, liver index, and body weight across and within the groups (**Table 4**). However, TG and cholesterol levels in liver tissue and serum were elevated in all the groups. Furthermore, glucose tolerance tests revealed that all the mouse models subjected to hypoxia had a significant increase in the area under the curve of Intraperitoneal Glucose Tolerance Test (IPGTT) and in the fasting glucose levels (**Supplementary Figure 3**; **Figure 2C**).

**Table 3:** Histologic characteristics of the established NAFLD and NASH mice (n=5 for each group).

Parameters	Control		NAFLD		C-NASH		M-NASH	
	Normoxia	Hypoxia	Normoxia	Hypoxia	Normoxia	Hypoxia	Normoxia	Hypoxia
Steatosis grade (0-3)	0	0	1.28 ± 0.42^#^	1.12 ± 0.34^#^	2.13 ± 0.37^#^	2.24 ± 0.38^#^	2.73 ± 0.32^#^	2.84 ± 0.36^#^
Lobular inflammation (0-3)	0	0.13 ± 0.10	0.16 ± 0.03	0.38 ± 0.12^#^	0.79 ± 0.22^#^	1.34 ± 0.28^*#^	1.06 ± 0.26^#^	1.95 ± 0.18^*#^
Ballooning score (0-2)	0	0	1.00 ± 0.00^#^	1.52 ± 0.34^*#^	2.00 ± 0.00^#^	2.00 ± 0.00^#^	2.00 ± 0.00^#^	2.00 ± 0.00^#^
NAS grade (0-8)	0	0.13 ± 0.10	2.38 ± 0.72^#^	2.97 ± 0.83^#^	5.08 ± 1.24^#^	5.64 ± 0.62^#^	5.71 ± 1.16^#^	6.28 ± 0.52^#^
Portal inflammation (0-3)	0	0	0.16 ± 0.03	0.28 ± 0.12^#^	0.59 ± 0.12^#^	0.94 ± 0.11^*#^	1.46 ± 0.1^#^	1.95 ± 0.12^*#^
Fibrosis stage (0-4)	0	0	0.11 ± 0.02	0.58 ± 0.05^*#^	1.12 ± 0.24^#^	2.23 ± 0.36^*#^	2.28 ± 0.18^#^	3.02 ± 0.42^*#^

On-way analysis of variance: *p < 0.05 between the two groups under the same conditions; ^#^p < 0.05 vs control group.

**Table 4:** General characteristics of the NAFLD and NASH mice (n=5 for each group).

Parameters	Control		NAFLD		C-NASH		M-NASH	
	Normoxia	Hypoxia	Normoxia	Hypoxia	Normoxia	Hypoxia	Normoxia	Hypoxia
Weight (g)	22.02 ± 0.63	25.74 ± 0.58	27.05 ± 4.15^#^	25.81 ± 1.77	30.77 ± 2.52^#^	26.83 ± 0.63^#^	12.38 ± 0.36^#^	16.77 ± 0.69^#^
FGB (mmol/L)	5.73 ± 0.25	6.77 ± 0.64	5.07 ± 0.35	12.97 ± 1.82^*#^	6.20 ± 1.11	10.23 ± 1.38^*#^	4.47 ± 0.67^#^	6.53 ± 2.06
Hemoglobin (mg/L)	189.25 ± 6.85	222.25 ± 24.98	105.00 ± 8.54^#^	145.25 ± 7.68^*#^	167.67 ± 2.08	231.75 ± 13.38^*#^	65.67 ± 24.58^#^	109.75 ± 16.82^*#^
Liver Index	0.03 ± 0.00	0.03 ± 0.00	0.04 ± 0.00^#^	0.04 ± 0.00^#^	0.04 ± 0.00^#^	0.05 ± 0.00^*#^	0.04 ± 0.00^#^	0.05 ± 0.00^*#^
Adenosine triphosphate (μmol/L)	1.60 ± 0.10	1.35 ± 0.12^*#^	1.79 ± 0.02^#^	1.63 ± 0.07^*^	1.96 ± 0.10^#^	1.79 ± 0.07^*#^	1.83 ± 0.12^#^	1.42 ± 0.06^*^
Triglycerides (liver)(U/L)	3.50 ± 0.12	3.39 ± 0.38	4.20 ± 0.64	3.50 ± 0.06	9.81 ± 0.56^#^	11.98 ± 0.84^*#^	9.23 ± 0.13^#^	10.44 ± 0.48^*#^
Cholesterol (liver) (U/L)	15.80 ± 3.06	17.98 ± 2.28	61.75 ± 5.50^#^	55.70 ± 8.08^#^	43.66 ± 7.17^#^	59.44 ± 12.34^*#^	33.67 ± 3.77^#^	37.70 ± 12.45^#^
Triglycerides (serum)(μmol/L)	0.62 ± 0.02	0.68 ± 0.04	0.85 ± 0.25	0.77 ± 0.19	0.94 ± 0.09	1.61 ± 0.42^*#^	1.16 ± 0.21^#^	1.37 ± 0.21^#^
Cholesterol (serum) (μmol/L)	0.79 ± 0.15	0.95 ± 0.13	2.94 ± 0.94^#^	3.07 ± 0.55^#^	2.07 ± 0.33^#^	3.59 ± 1.13^*#^	1.55 ± 0.25	1.89 ± 0.60^#^
ALT (serum) (U/L)	25.67 ± 3.82	31.88 ± 1.93	52.90 ± 5.57^#^	80.11 ± 6.02^*#^	116.63 ± 4.96^#^	161.51 ± 9.91^*#^	95.26 ± 7.11^#^	140.34 ± 11.32^*#^
AST (serum) (U/L)	27.31 ± 2.85	31.99 ± 1.77	52.36 ± 3.80^#^	65.85 ± 4.78^*#^	113.74 ± 4.21^#^	149.31 ± 4.63^*#^	129.39 ± 4.98^#^	165.42 ± 5.11^*#^

On-way analysis of variance: *p < 0.05 between the two groups under the same conditions; ^#^p < 0.05 vs control group.

We then examined HIF expression levels after chronic hypoxia. In contrast to HIF-1α expression levels, HIF-2α expression levels were significantly increased in all groups that had been subjected to hypoxia compared with those in the control group. Furthermore, we observed a significant increase in myofibroblast markers, α-SMA and Col1A in HSCs obtained from groups that had been subjected to hypoxia. The M-NASH group exhibited the highest increase, suggesting enhanced severity compared with the C-NASH group (**Supplementary Figure 4**; **Figures 2D–J**).

To understand the effect of chronic hypoxia on glutaminolysis in HSCs, the expression of two key enzymes, GLS1 (which decomposes glutamine) and SlC38A1 (which facilitates glutamine transfer) in the C-NASH and M-NASH groups was quantified by qRT-PCR (**Figures 2K, L**). The mRNA levels for both enzymes showed a significant increase in these two groups in response to hypoxia. Moreover, YAP1 protein levels increased in all groups subjected to hypoxia compared with those in the normoxic group, whereas phosphorylated p-YAP(S127) expression significantly decreased. These findings corroborate our observations in human patients, underlying the potential influence of hypoxia on fatty liver disease progression.

### Hypoxia impaired hepatic mitochondria, inhibited oxidative phosphorylation, and increased ROS production in mouse models for NAFLD/NASH

**Figure 3:**
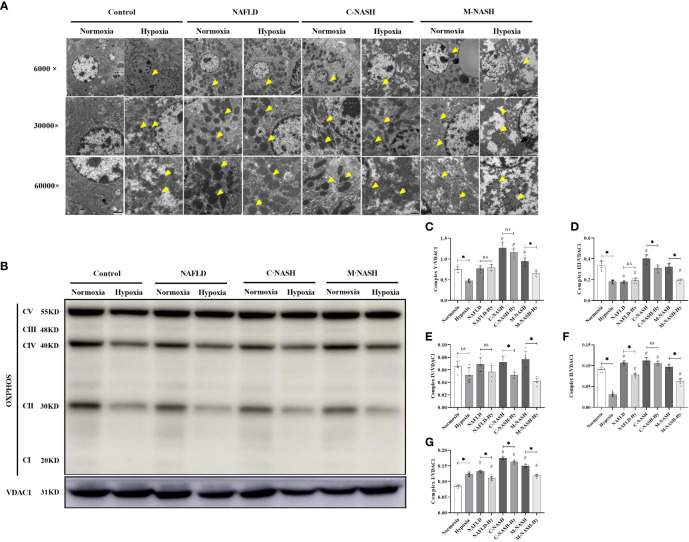
Chronic hypoxia impaired hepatic mitochondria and inhibited the oxidative phosphorylation complex in mouse models for NAFLD and NASH. **(A)** Representative TEM images showing the morphology of mitochondria in liver samples from mouse models for NAFLD and NASH. Samples from animals subjected to hypoxia showed enlarged and swollen mitochondria with disordered cristae. **(B)** Image of western blot from mouse liver lysates showing bands corresponding to OXPHOS complexes, with β-Tubulin used as the loading control. **(C–G)** Quantification of band densities for OXPHOS complexes I, II, III, IV, and V. The samples corresponding to animals subjected to hypoxia showed decreased expression for all OXPHOS complexes (n = 5; *p < 0.05 between the two groups under the same conditions, ^#^p < 0.05 vs Control group). TEM, Transmission electron microscopy; NAFLD, Non-alcoholic fatty liver disease; NASH, Non-alcoholic hepatitis; OXPHOS, oxidative phosphorylation. ns: No significance.

Given the importance of mitochondrial dysfunction in NAFLD progression, we investigated the impact of chronic hypoxia on mitochondrial function. TEM revealed no significant differences in mitochondrial morphology between the normoxic and hypoxic groups. However, in the hypoxic NAFLD group, we observed swollen mitochondria with irregular edges, indicative of severe damage under hypoxic conditions. This mitochondrial damage was more severe in the C-NASH and M-NASH groups, with significant swelling, fusion, and cristae disappearance in the mitochondria following hypoxia (**Figure 3A**). We also observed a reduction in the expression of the oxidative phosphorylation complex, which is integral to mitochondrial oxidative metabolism. **Figure 3B**; **Supplementary Figure 5** shows a significant decrease in complex I expression in the hypoxic group, with similar trends observed for complexes II, IV, and V. The most evident decreases after low oxygen treatment were observed in the M-NASH and C-NASH groups. We also found that ATP production was significantly decreased in all the experimental groups in response to hypoxia (**Figures 3C–G**).

**Figure 4:**
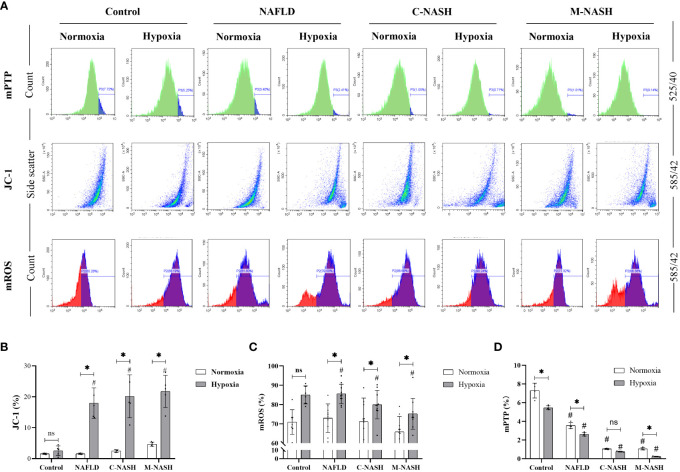
Chronic hypoxia augmented mitochondrial ROS production by impairing mitochondrial membrane and opening mPTPs in hepatocytes from mouse models for NAFLD and NASH. **(A)** Flow cytometry images and visualisations of the ΔΨm, ROS, and mPTP opening. Mice subjected to hypoxia showed decreased ΔΨm, increased ROS production, and increased mPTP opening. **(B)** Quantification of JC-1 levels of ΔΨm. Hypoxic mice showed a significantly decreased ΔΨm. (n = 5; *p < 0.05 between the two groups under the same conditions, ^#^p < 0.05 vs Control group). **(C)** Quantification of mitochondrial ROS levels. Hypoxic mice showed significantly increased ROS production. (n = 5; *p < 0.05 between the two groups under the same conditions, ^#^p < 0.05 vs Control group). **(D)** Quantification of mPTP opening. Mice subjected to hypoxia showed a significant increase in mPTP opening (n = 5; *p < 0.05 between the two groups under the same conditions, ^#^p < 0.05 vs Control group). ROS, Reactive oxygen species; mPTP, Mitochondrial permeability transition pore; NAFLD, Non-alcoholic fatty liver disease; NASH, Non-alcoholic hepatitis; ΔΨm, mitochondrial membrane potential.ns: No significance.

We hypothesised that ROS production in the mitochondria of liver cells and mitochondrial membrane damage would significantly increase under hypoxic conditions. Hepatocytes were isolated and flow cytometry was applied to measure JC-1, mROS, and mPTP, all of which are related to mitochondrial activity and ROS production. The results showed that the JC-1 and mROS detection indices were significantly increased in the NAFLD and NASH groups subjected to hypoxia; however, the higher amount of mPTP was significantly decreased in these experimental groups (**Figures 4A–D**).

### HIF-2α upregulation increased myofibroblasts dependent on YAP-induced glutaminolysis *in vitro*

**Figure 5:**
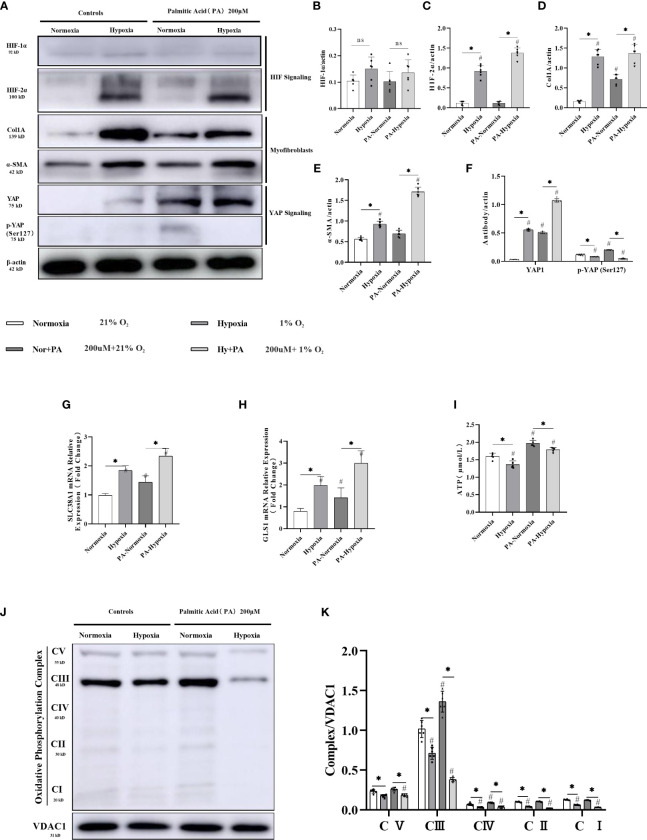
HIF-2α overexpression triggered by hypoxia augmented YAP-induced myofibroblast differentiation and inhibited the mitochondrial oxidative phosphorylation complex, and ATP production in LX-2 cells. **(A)** Western blot results for fibrosis-related proteins from LX-2 cell lysates, with β-actin as loading control. Cell cultures subjected to hypoxia showed increased expression of HIF-2α, α-SMA, YAP1, and Col1A, and decreased phosphorylation of YAP (Ser127). **(B–F)** Quantification of western blot results confirmed increased expression of HIF-1α, HIF-2α, α-SMA, Col1A, YAP1, and decreased expression of p-YAP (Ser127) in hypoxic cultures. (n = 3; *p < 0.05 between the two groups under the same conditions, ^#^p < 0.05 vs normoxia group). The mRNA expression levels of SLC38A1 **(G)** and GLS1 **(H)** were increased in hypoxic cultures. (n = 3; *p < 0.05 between the two groups under the same conditions, ^#^p < 0.05 vs normoxia group). **(I)** ATP production was decreased in cells subjected to hypoxia. (n = 3; *p < 0.05 between the two groups under the same conditions, ^#^p < 0.05 vs normoxia group). **(J)** Western blot results for oxidative phosphorylation proteins from LX-2 cell lysates, with VDAC1 as loading control. Cell cultures subjected to hypoxia showed decreased expression of all OXPHOS complexes. **(K)** Quantification of western blot results confirmed decreased expression of all OXPHOS complexes in hypoxic cultures (n = 3; *p < 0.05 between the two groups under the same conditions, ^#^p < 0.05 vs normoxia group). YAP1, Yes-associated protein; HIF-1α, Hypoxia-inducible factor-1α; HIF-2α, Hypoxia-inducible factor-2α; Col1A, Collagen type I alpha 1; α-SMA, α-smooth muscle actin; p-YAP(S127), Phosphorylated yes-associated protein; GLS1, Glutaminase 1; OXPHOS, oxidative phosphorylation. ns: No significance.

HIF-2α, α-SMA, and Col1A showed increased expression in LX-2 cell cultures subjected to hypoxia, with the highest increase observed in cultures supplemented with palmitate (**Supplementary Figure 6**; **Figures 5A–F**). qPCR detection further confirmed two-fold or higher increases in the expression of SLC38A1 and GLS1 (**Figures 5G, H**). These pieces of evidence from *in vitro* cell culture support the hypothesis that hypoxia exacerbates liver fibrosis. The trends in the changes observed in the expression levels of HIF-2α, α-SMA, Col1A, and the mitochondrial oxidative phosphorylation complex (**Supplementary Figure 6**; **Figures 5J, K**), as well as those in ATP production (**Figure 5I**) were consistent with those observed *in vivo* in the mouse models, suggesting that hypoxia-induced upregulation of HIF-2α promotes cell fibrosis via the YAP1 and mitochondrial energy metabolisms.

**Figure 6:**
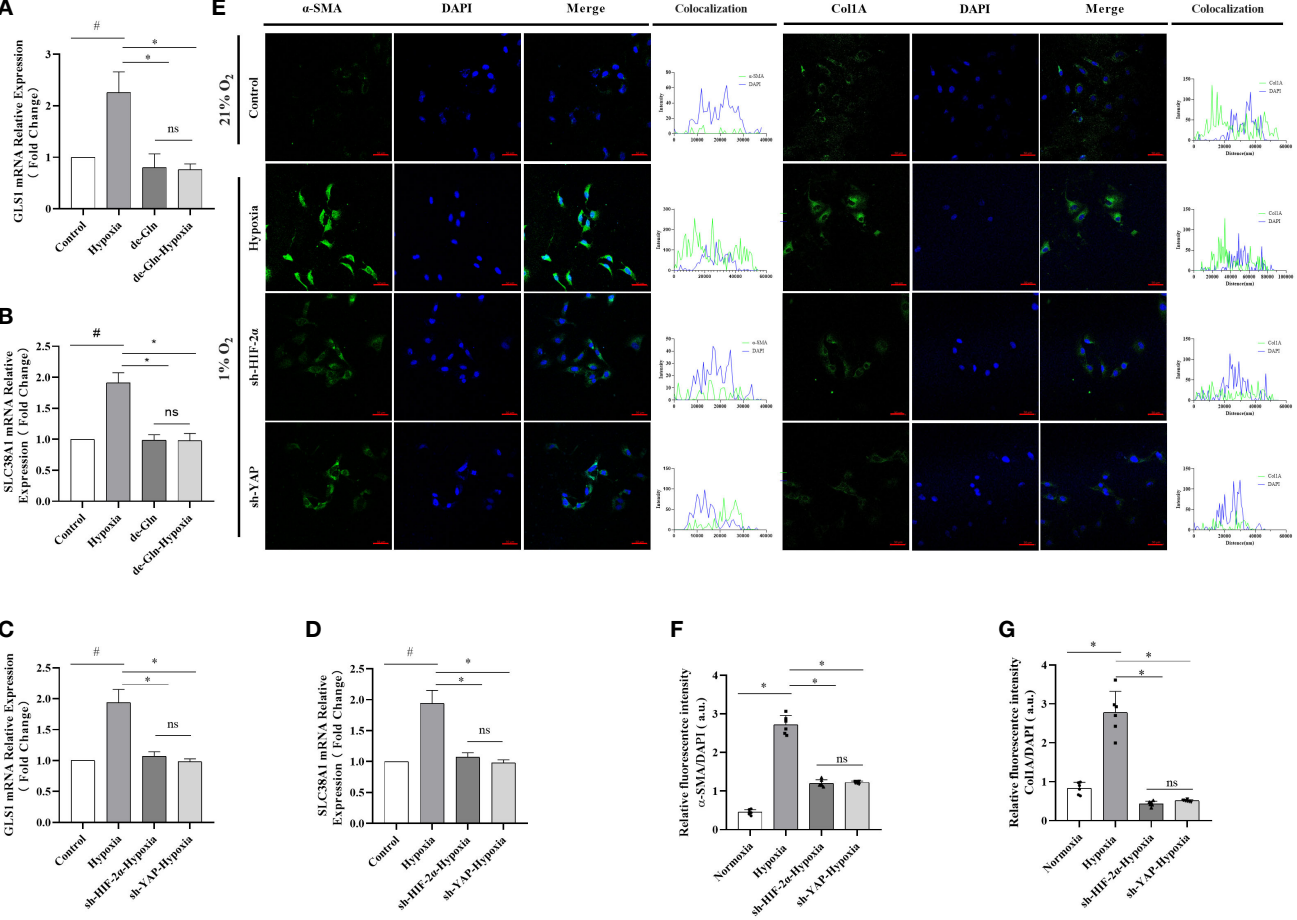
HIF-2α overexpression triggered by hypoxia augmented YAP-induced myofibroblast differentiation in a glutamine-dependent manner, which was abolished by the downregulation of HIF-2α and YAP in LX-2 cells. **(A)** GLS1 mRNA expression in LX-2 cells under different conditions. Hypoxia increased GLS1 expression, which was abolished by HIF-2α and YAP1 downregulation (n = 3; *p < 0.05 between the two groups under the same conditions, #p < 0.05 vs control group). **(B)** SLC38A1 mRNA expression in LX-2 cells under different conditions. Hypoxia increased SLC38A1 expression, which was abolished by HIF-2α and YAP1 downregulation (n = 3; *p < 0.05 between the two groups under the same conditions, ^#^p < 0.05 vs control group). **(C)** GLS1 mRNA expression in LX-2 cells after HIF-2α and YAP1 downregulation. HIF-2α and YAP1 downregulation abolished the hypoxia-induced increase in GLS1 expression (n = 3; *p < 0.05 between the two groups under the same conditions, ^#^p < 0.05 vs control group). **(D)** SLC38A1 mRNA expression in LX-2 cells after HIF-2α and YAP1 knockdown. HIF-2α and YAP1 knockdown abolished the hypoxia-induced increase in SLC38A1 expression (n = 3; *p < 0.05 between the two groups under the same conditions, ^#^p < 0.05 vs control group). **(E)** Immunofluorescence staining with Alexa Fluor 488 (green) for α-SMA and Col1A in LX-2 cells, and colocalization with DAPI (blue). **(F)** Quantification of α-SMA expression. Hypoxia increased α-SMA expression, and this was abolished by HIF-2α and YAP1 downregulation (n = 3; *p < 0.05 between the two groups under the same conditions, ^#^p < 0.05 vs control group). **(G)** Quantification of Col1a expression. Hypoxia increased Col1A expression, which was abolished by HIF-2α and YAP1 downregulation (n = 3; *p < 0.05 between the two groups under the same conditions, ^#^p < 0.05 vs control group). Scale bar: 50 μm. Similar results were obtained from three independent experiments, and representative photographs are shown in each case. Data are presented as means ± SD (n=3). GLS1, Glutaminase 1; YAP1, Yes-associated protein; HIF-2α, Hypoxia-inducible factor-2α; Col1A, Collagen type I alpha 1; α-SMA, α-smooth muscle actin. ns: No significance.

To confirm that glutamine is an energy source for myofibroblasts, LX-2 cell cultures were exposed to 1% O_2_ concentrations in a hypoxic chamber for three days using either standard or glutamine-deprived glucose medium. However, no significant differences in cell viability were observed between the hypoxia and control groups when glutamine-deprived culture medium was used (**Figures 6A, B**). Cell migration ratios were significantly decreased when glutamine-deprived glucose medium was used (**Supplementary Figures 7A, B**), and protein levels of both α-SMA and Col1A were downregulated (**Supplementary Figures 7C, D**). Interestingly, after HIF-2α and YAP were experimentally downregulated via shRNAs, upregulation in the expression levels of GlS1 and GlS2, which are related to glutamine metabolism during hypoxia, was no longer evident, and the fluorescence signal was completely reversed. Moreover, the expression levels of GLS1 and SLC38A1 were significantly reduced in the hypoxia group (**Figures 6C, D**). We also observed an increase in active myofibroblastic phenotypes, as demonstrated by α-SMA immunofluorescence, in the hypoxia group (**Figure 6E** and quantified in **Figures 6F, G**). Additionally, glutaminolytic activity was higher in the group exposed to 1% O_2_ compared with that in the control group (exposed to 21% O_2_).The fluorescence intensity of α-SMA and Col1A remained low, and mRNA expression levels for GLS1 and SLC38A1 were lower than those in the control group.

### HIF-2α enhanced activities and myofibroblasts in HSCs by inhibiting YAP phosphorylation

**Figure 7:**
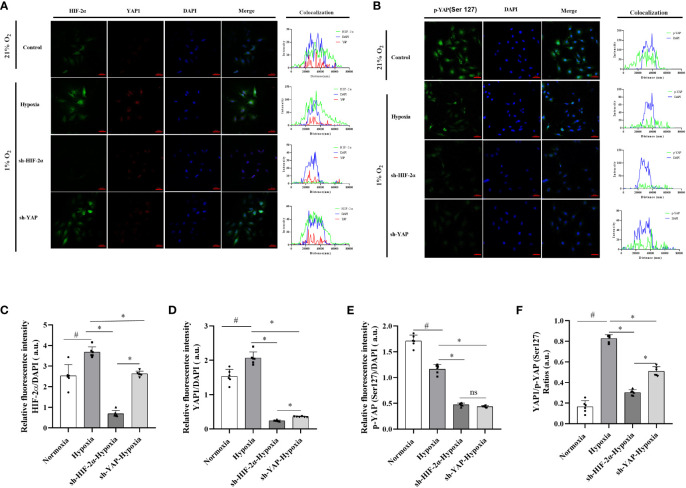
Expression levels and distribution of YAP1 and p-YAP (Ser 127) in LX-2 cells after HIF-2α and YAP1 knockdown. **(A)** LX-2 cells stained with DAPI to visualise nuclei (blue) and with antibody-conjugated Alexa Fluor 488 or Alexa Fluor 647 to visualise the distribution of HIF-2α (green) and YAP1 (red), respectively. **(B)** LX-2 cells stained with DAPI to visualise nuclei (blue) and antibody-conjugated Alexa Fluor 488 to visualise the distribution of p-YAP (Ser 127) (green). **(C)** Quantification of HIF-2α immunofluorescence staining relative to DAPI intensity in LX-2 cells. **(D)** Quantification of YAP1 immunofluorescence staining relative to DAPI intensity in LX-2 cells. **(E)** Quantification of p-YAP(S127) immunofluorescence staining relative to DAPI intensity in LX-2 cells. **(F)** Analysis of immunofluorescence staining intensity ratios between YAP1 and p-YAP (Ser 127). Scale bar: 50 μm. Similar results were obtained from three independent experiments, and representative photographs are shown in each case. Data are presented as means ± SD (n=3). *P<0.05, NS: P>0.05 compared with the same conditions. ^#^P<0.05 compared with the control group. YAP1, Yes-associated protein; p-YAP(S127), Phosphorylated yes-associated protein; HIF-2α, Hypoxia-inducible factor-2α.

HIF-2α is highly expressed in LX-2 cells in response to chronic hypoxia, promoting glutamine catabolism via the YAP signalling pathway and thus allowing the cells to obtain the energy required for activation, proliferation, and differentiation into myofibroblasts. We observed that, similarly to those in the control group, the YAP fluorescence signal was weakly distributed in the cytoplasm of the cells in the hypoxia (1% O_2_) group. However, the ratio of YAP1 and p-YAP(S127) exhibited no changes in the YAP knockdown group (**Supplementary Figure 8**), even after hypoxia. When HIF-2α was experimentally downregulated (**Supplementary Figure 9**), analysis of the fluorescence signal showed that under hypoxic conditions (1% O_2_), YAP fluorescence was highly distributed in the cytoplasm of the cells, indicating an increase in p-YAP(S127). However, the region of overlap between the YAP fluorescence signal and DAPI fluorescence was significantly reduced, indicating a reduction in YAP1 levels (**Figures 7A, C, D**). In addition, the ratios of YAP1 and p-YAP (Ser127) were significantly reduced (**Figures 7B, E**). These findings imply a mechanism in which HIF-2α upregulation inhibits p-YAP(S127) (**Figure 7F**). This leads to nuclear translocation of the YAP mRNA, inducing glutaminolysis in HSCs subjected to chronic hypoxia.

## Discussion

Hypoxia is associated with the development and progression of NAFLD to NASH (18, 34, 35). However, the underlying mechanisms are unknown. Novel mechanisms through which chronic hypoxia activates HIF-2α, affecting glutamine catabolism in HSC-derived myofibroblasts, that were found in this study include: (1) Direct enhancement of fibrosis by HIF-2α in NASH through increased glutaminolysis; (2) Inhibition of mitochondrial activity in hepatocytes (but enhancement in HSCs) by HIF-2α; (3) Increase in GLS1 expression in HSCs by HIF-2α, mediated through YAP; and (4) Inhibition of p-YAP but enhancement of YAP by HIF-2α, facilitating YAP nuclear transfer, interaction with TAZ, and GLS1 mRNA expression.

The proteins that control NASH progression under chronic hypoxia are unknown. To circumvent the effect of different genetic backgrounds while investigating the potential driving role of chronic hypoxia in NAFLD and NASH progression, we have chosen NAFLD/NASH patients of Han Chinese ethnicity living in altitudes above 2300m as our study population. Exposure to chronic hypoxaemia was confirmed in the participants of our study by their elevated haemoglobin levels (36).

Several studies have revealed that both HIF-1α and HIF-2α (11, 37) control the metabolism of reprogrammed hepatocytes (38, 39). HIF-1α and HIF-2α recognise and bind hypoxia response elements to activate the cellular response to hypoxia. However, HIF-1α and HIF-2α have different structures and expression patterns (40–42). In this study, HIF-2α displayed different expression levels and distribution in hepatic tissue from both patients with NASH and mouse models for NASH subjected to persistent hypoxia, compared with those of HIF-1α. Moreover, treatment with an HIF-2α agonist improved mice with NASH and alleviated fibrotic NASH development. This is because HIF-1a influences angiogenesis and regulates endothelial function in intermittent hypoxia (43, 44) rather than in chronic and persistent hypoxia in fatty liver diseases (45–48). Moreover, HIF-2α is involved in lipid and amino acid metabolism in NAFLD, which may play a pivotal role in disease progression and be a major potential therapeutic target (49). In this study, we also found that HIF-2α was highly expressed in patients and mouse models for NASH exposed to chronic hypoxia. HIF-2α may be recruited to promote collagen and ECM accumulation, and activate HSCs to differentiate into myofibroblasts and thus enhance fibrosis (39, 50). HIF-2α showed fibrosis advances in chronic kidney diseases with chronic hypoxia (51, 52). We observed increased HIF-2α expression in mice with NASH and in LX-2 cell cultures. Identifying whether HIF-2α plays a role in the reprogramming of hepatocyte and HSC metabolism is crucial.

Chen et al. and Evert et al., 1998 revealed heightened l activity of oxidative phosphorylation in the mitochondria of cells cultured *in vitro*, but not in fibrotic livers *in vivo*. Moreover, we used TEM and western blot analysis to assess the impact of chronic hypoxia on the mitochondria of LX-2 cells and in mice with NASH. We found decreased mitochondrial numbers, suppressed mitochondrial activity, and decreased mitochondrial membrane potential and ATP production, both *in vivo* and *in vitro*, which demonstrates that upregulated HIF-2α significantly inhibits mitochondrial activity. We further investigated ROS production in mitochondria from liver tissue and from those in LX-2 cells. We found that mPTPs in the mitochondrial inner membrane opened more in response to calcium overload in the mitochondrial matrix when HIF-2α was upregulated, and that this effect can be reversed by HIF-2α downregulation in LX-2 cells. Therefore, mPTP opening, decreased mitochondrial activity, and increased ROS production are closely associated with the progression of NAFLD and NASH after HIF-2α upregulation (53, 54).

HSCs contribute to hepatic fibrosis in NAFLD or NASH by becoming activated and differentiating into myofibroblasts, which secrete extracellular matrix (7, 8). This requires additional energy, obtained from central carbon metabolism (9, 10, 55, 56). a-SMA significantly enhances HSC-associated fibrosis and myofibroblast differentiation. Fibrosis, collagen secretion, and differentiation of HSCs into myofibroblasts have been reported to significantly decrease in response to HIF-2α downregulation (57). Therefore, chronic hypoxia significantly exacerbates the progression of NAFLD and NASH by an HIF-2α-induced mechanism of activation and differentiation of HSCs into myofibroblasts.

Determining whether HIF-2α has the same effects on mitochondrial activity in hepatocytes and HSCs would be useful to understand the progression of NAFLD to NASH. We discovered that HIF-2α inhibited mitochondrial ATP production and reduced hepatocyte numbers, while highly increasing ROS production in the mitochondria. However, an increase in mitochondrial respiratory function, including ATP production, has been reported in the case of HSCs (58). ROS are generated in excess by impaired mitochondria and are a key factor on NASH pathogenesis (59–62). Fluorescent detectors have been used to show that ROS levels in the mitochondria are markedly increased under hypoxic conditions (53, 54). In this study, we confirmed that mPTP opening gradually increased according to the degree of fibrosis present in mice with NASH. These findings indicate that hypoxia positively regulates ROS production by inhibiting mitochondrial function (63). Hypoxia suppresses mitochondrial function and induces ROS production in large quantities, which is essential for collagen accumulation and ECM remodelling (64).

Furthermore, we discovered a link between GLS1 overexpression in HSCs and increased ATP production. In particular, we detected that glutamine levels were low in the plasma of the NASH patients, but that it was enriched in glutamate, and that HIF-2α levels in liver tissue were enhanced. Glutamine catabolism may provide the additional energy required by HSCs during chronic hypoxia (28). We found that GLS1 levels increased in both patients and in an animal model for NASH, and that this was dependent on fibrosis, suggesting that GLS1 may enhance glutaminolysis associated with hypoxia-induced HIF-2α (37, 65). Further research using LX-2 cells *in vitro* could be helpful to understand the relationship between HIF-2α and glutaminolysis.

The metabolic reprogramming that controls HSC activation from a quiescent status in response to changes in the microenvironment has only garnered attention recently (16). We propose that glutamine, as the largest non-essential amino acid present in plasma, could be used to replenish the intermediates of the tricarboxylic acid cycle and thus generate nucleotides and fatty acids. We found increased mitochondrial respiration in zone 3. Moreover, we found increased mitochondrial fusion rather than fission when we assessed morphological changes, which could be attributed to decreased mitochondrial turnover to compensate for oxygen deprivation during hypoxia. Glutamine metabolism, either oxidative or reductive, occurs in mitochondria (66, 67). We found increased expression of GLS1 and GDH, suggesting increased oxidative reactions. HIF-2α may be involved in the activation of the oxidative pathways; however, further experiments are needed to characterise glutaminolysis taking place in the mitochondria. HIF-2α in hypoxic liver tissues increased SNAT expression in the cell membrane of HSCs, prompting more glutamine translocation into the cells (28, 68–70).

We studied HSCs to identify the role of HIF-2α in myofibroblast differentiation leading to NASH. This study found that increased GLS1 expression enhanced HSC glutaminolysis. The molecular pathways regulating glutaminolysis are mostly associated with Hippo and YAP signals; however, no direct evidence exists to verify an interaction between HIF-2α and YAP. Hypoxia also increased YAP levels in the nucleus but decreased those of phosphorylated YAP in the HSC cytosol. HIF-2α may stimulate the Hippo pathway and activate YAP to enhance nuclear translocation and thus upregulate GLS1 mRNA expression. We also found that decreased YAP levels did not change a-SMA levels without affecting HIF-2α expression, which indicates that YAP-induced glutaminolysis is HIF-2α-dependent, and HIF-2α regulates YAP expression and decreases p-YAP(S127) levels in HSCs. Moreover, we found that HIF-2α inhibited YAP phosphorylation at serine 127, which was consistently identified by genetic HIF-2α knock-out mice, to suppress hepatocellular carcinoma invasion and proliferation by inhibiting YAP through AKT activation (71). This is supported by the presence of the adhesive junction protein α-catenin in YAP serine 127 (72, 73), which can be phosphorylated by LAS T1/2, Hippo signal proteins, and recruited E3 ubiquitin ligases, leading to YAP/TAZ degradation and reduced nuclear translocation. HIF2α upregulates GLS1 expression and is involved in glutaminolysis. Our research results show that targeting HIF-2α regulation may serve as a new approach to alleviate hypoxic stress, interfere with the YAP signalling pathways, and prevent fibrosis progression in NAFLD.

However, our study does have some limitations, including limited clinical data, which underscores the need to increase the number of tissue samples. The exposure to hypoxia that the experimental animals were subjected to may not accurately reflect the specific hypoxia conditions affecting liver tissue, and gene manipulation may be required for *in vivo* validation. Despite the fact that three different animal models in our study, each of these models only partially represent the disease features of NAFLD and NASH, and thus cannot fully mirror all manifestations of these diseases. Moreover, it has been recently reported that circadian rhythm (74) and the endocrine system (75), are also involved in this phenomenon. This research focused only on HIF proteins involved in the hypoxia signalling pathway. Additionally, while glutamine catabolism is the main energy source for the myofibroblasts derived from HSCs in chronic liver disease, this study only considered the Hippo-YAP pathway, without conducting an omics analysis. Lastly, the use of the LX-2 cell line for the *in vitro* studies might not perfectly reflect the actual *in vivo* microenvironment of liver tissue.

## Conclusion

In summary, we found that HIF-2α enhances glutamine catabolism in myofibroblasts derived from HSCs and inhibits YAP phosphorylation, promoting fibrosis in NAFLD. Moreover, we have found that this pathway might serve as a potential target for interventions aimed at preventing the progression of NAFLD to NASH.

## Data availability statement

The original contributions presented in the study are included in the article/**Supplementary Material**. Further inquiries can be directed to the corresponding authors.

## Ethics statement

Ethical approval was not required for the studies on humans in accordance with the local legislation and institutional requirements because only commercially available established cell lines were used. The animal study was approved by Ethics Committee of the Medical College of Qinghai University. The study was conducted in accordance with the local legislation and institutional requirements.

## Author contributions

RY: Data curation, Methodology, Formal analysis, Investigation, Visualizaion, Software, Writing - review & editing. HC and XZ: Resources, Writing - review & editing. GB: Validation, Writing - review & editing. ZB: Data curation, Methodology, Supervision, Conceptualization, Formal analysis, Project administration, Validation, Funding acquisition, Visualizaion, Software, Writing - original draft. RG: Supervision, Writing - review & editing.
