# Supplementary material for: Hypoxia-inducible factor-2α promotes fibrosis in non-alcoholic fatty liver disease by enhancing glutamine catabolism and inhibiting yes-associated protein phosphorylation in hepatic stellate cells
Source: Front Endocrinol (Lausanne). 2024 Feb 28;15:1344971. doi: 10.3389/fendo.2024.1344971 (PMC10946064; doi:10.3389/fendo.2024.1344971)
Supplement: Supplementary file 1 [file DataSheet_1.pdf]

Figures: A total of 9 figures,   Tables: A total of 3 tables.

HIF-2α promotes fibrogenesis of nonalcoholic fatty liver diseases through enhanced glutamine catabolism in hepatic stellate cells via inhibiting phosphorylation of YAP

Fig. S1 Validation controls for GLS antibodies in immunohistochemistry, negative control has been made by the same procedures with the other two conditions but without the primary antibody, GLS1.

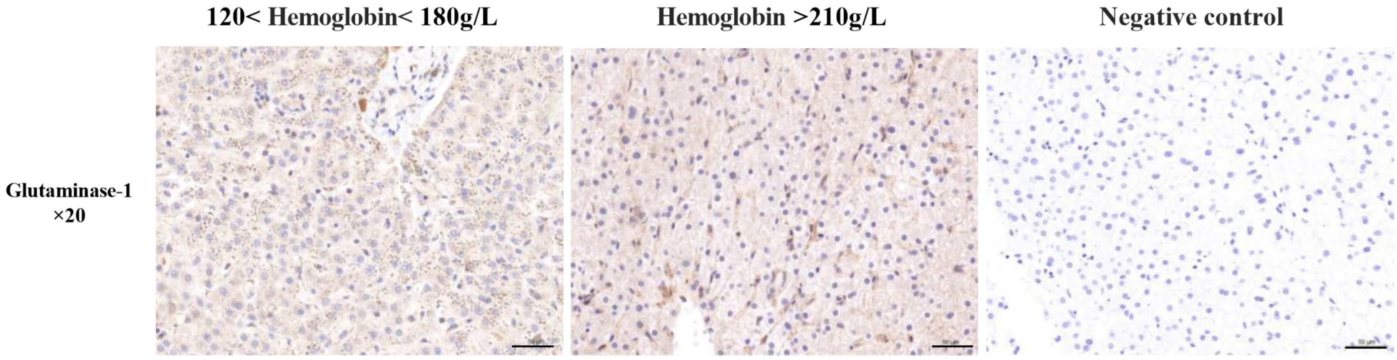

Fig. S2 Immunofluorescence of HIF-2α and α-SMA in livers from the murine models

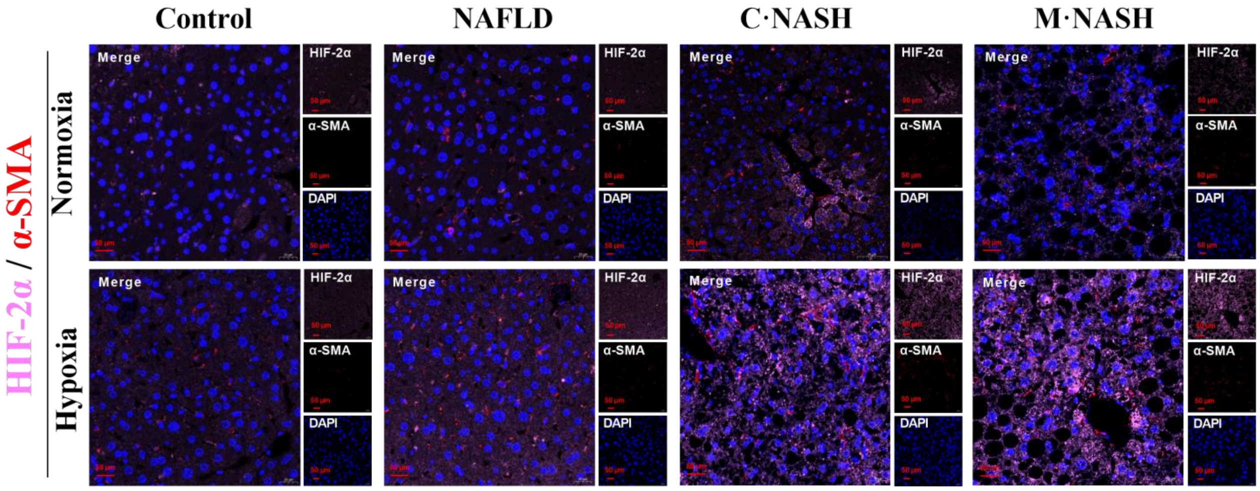

Immunofluorescence staining shows a significant increase in the expression of HIF-2α (pink) and α-SMA (red) in livers from the hypoxia murine models, and colocalization with DAPI (blue). (n = 5; magnification, 400×).

Fig. S3 Results of IPGTT experiment in NAFLD model mice

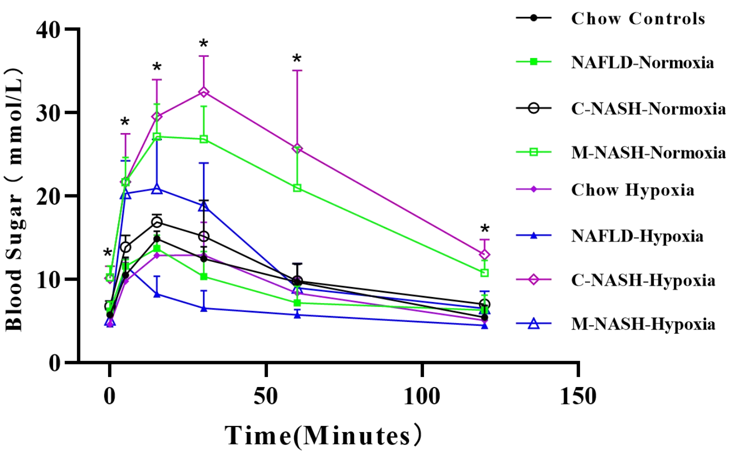

Fig. S4 Western blot strips for the original image of figure 2, which has been cut off and shown in the figure 2 in the manuscript

HIF-1-alpha antibody 92 kDa

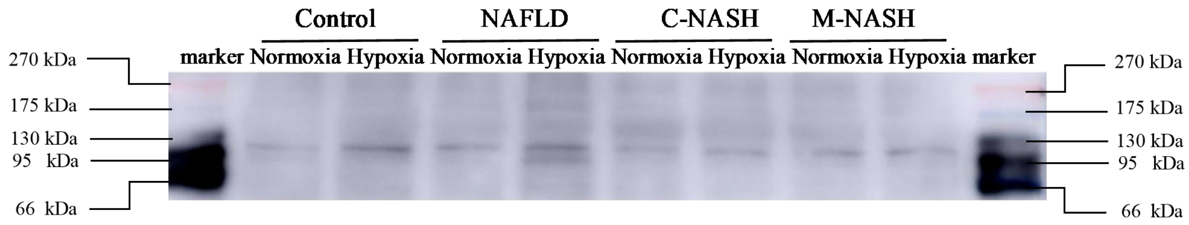

HIF-2-alpha antibody 100 kDa

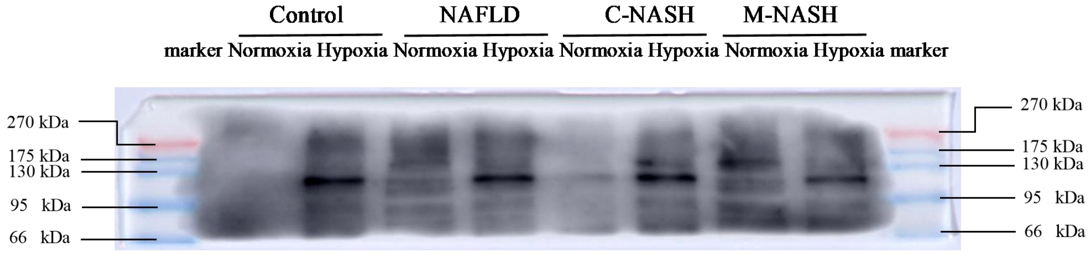

α-Smooth Muscle Actin 42kDa

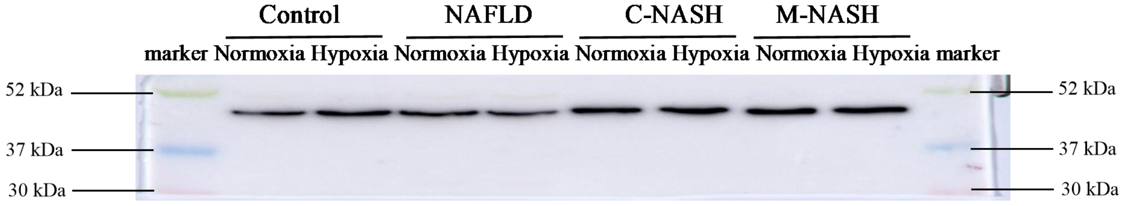

Collagen I antibody 139, 220 kDa

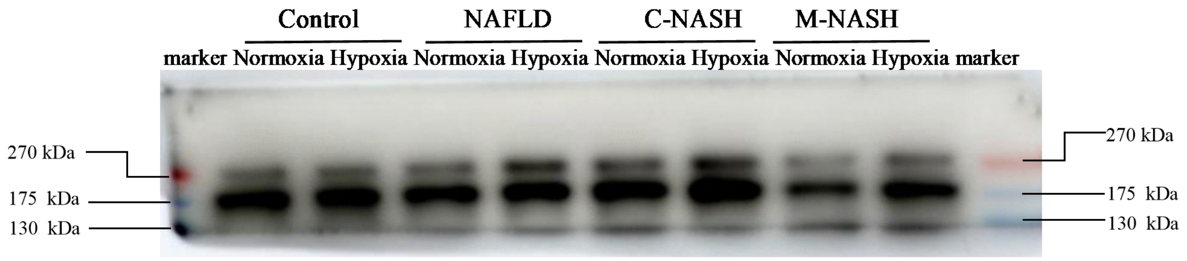

YAP1 antibody 75 kDa

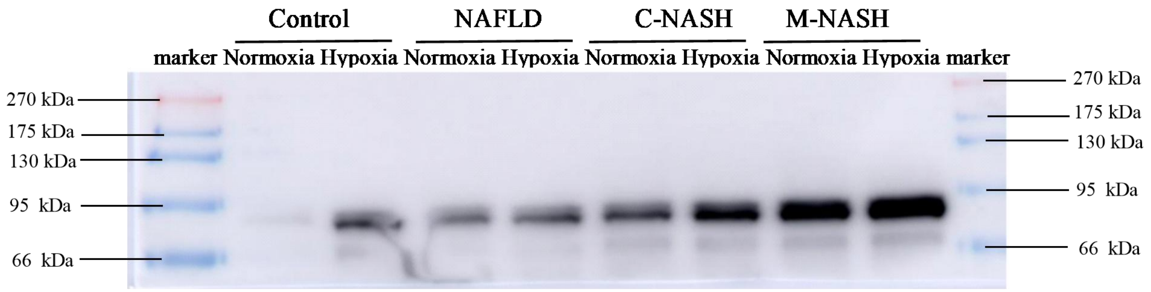

YAP1 (phospho S127) antibody 75 kDa

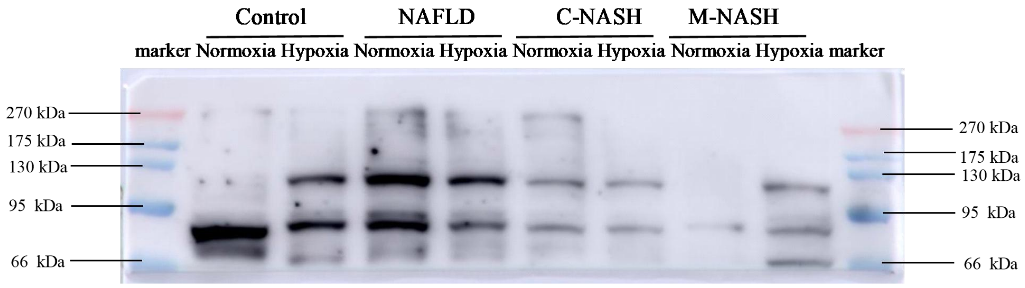

α/β-Tubulin Antibody 55 kDa

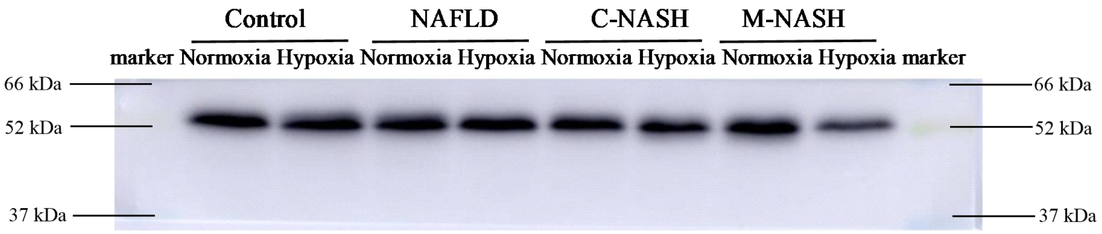

Fig. S5 Western blot strips for the original image of figure 3, which has been cut off and shown in the figure 3 in the manuscript

Total oxphosphate Rodent WB antibody Cocktails 20-55 kDa

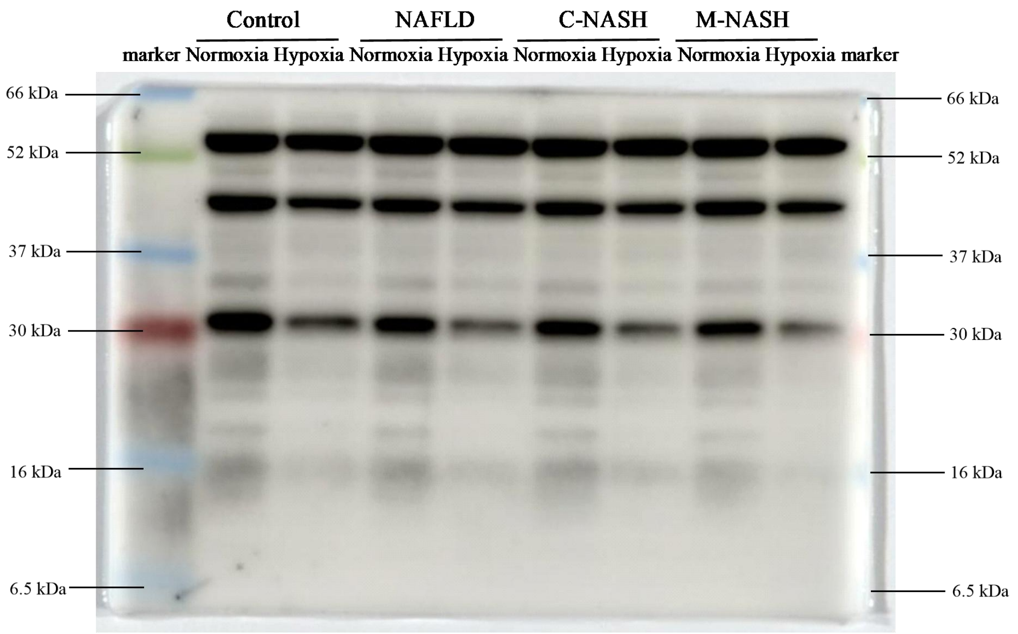

Anti-VDAC1/Porin Antibody 31 kDa

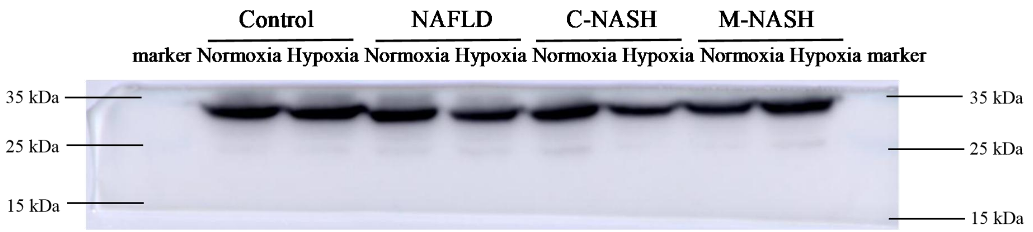

Fig. S6 Western blot strips for the original image of figure 5, which has been cut off and shown in the figure 5 in the manuscript

HIF-2-alpha antibody 100 kDa

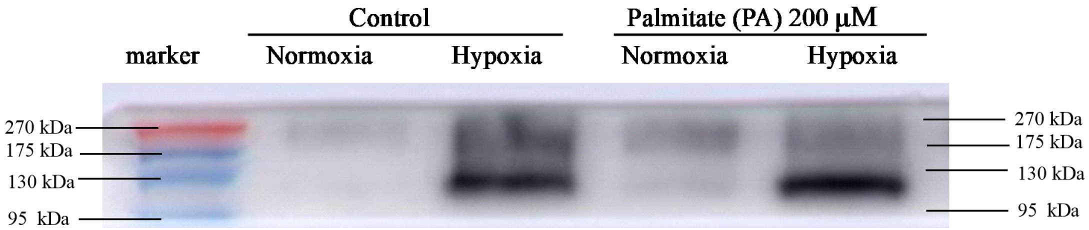

Collagen I antibody 139, 220 kDa

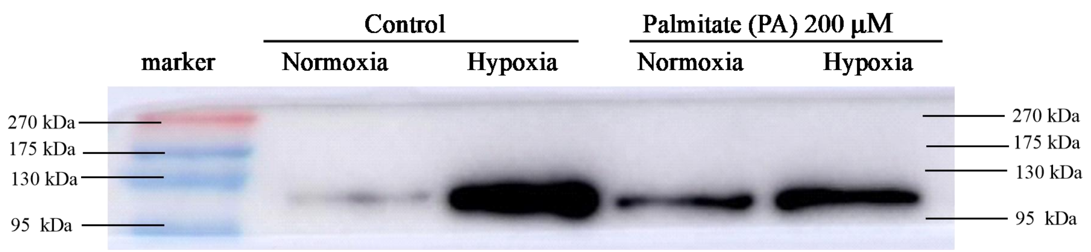

α-Smooth Muscle Actin 42kDa

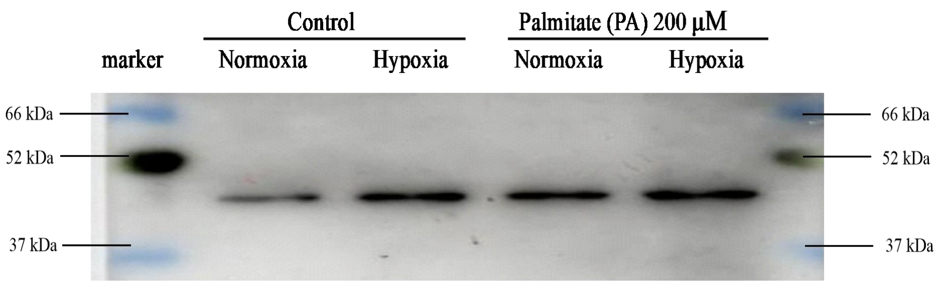

active YAP antibody 75 kDa

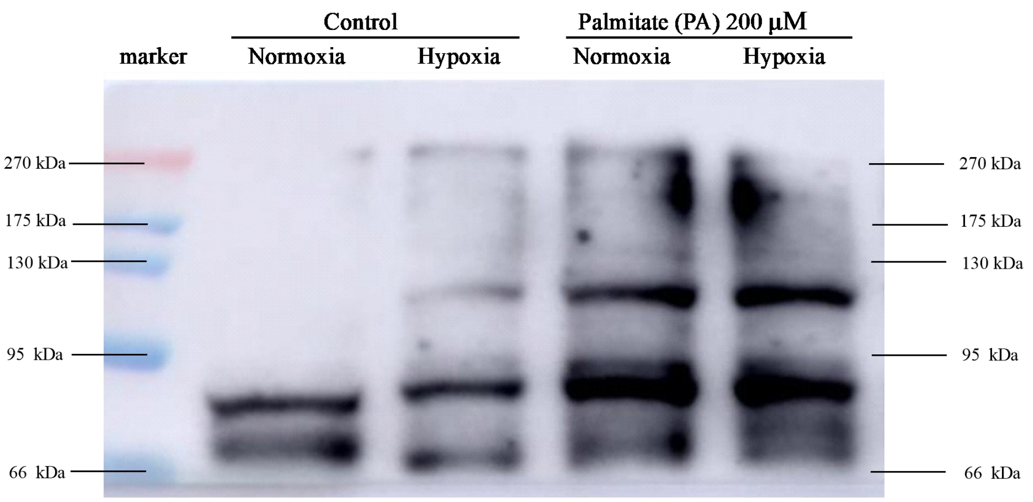

YAP1 (phospho S127) antibody 75 kDa

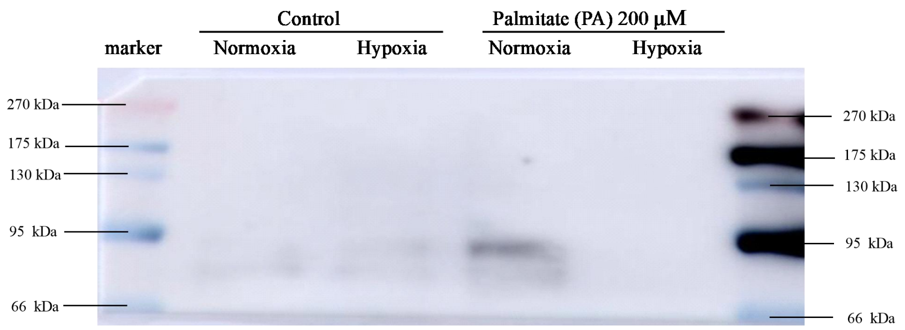

β-Actin Antibody 42kDa

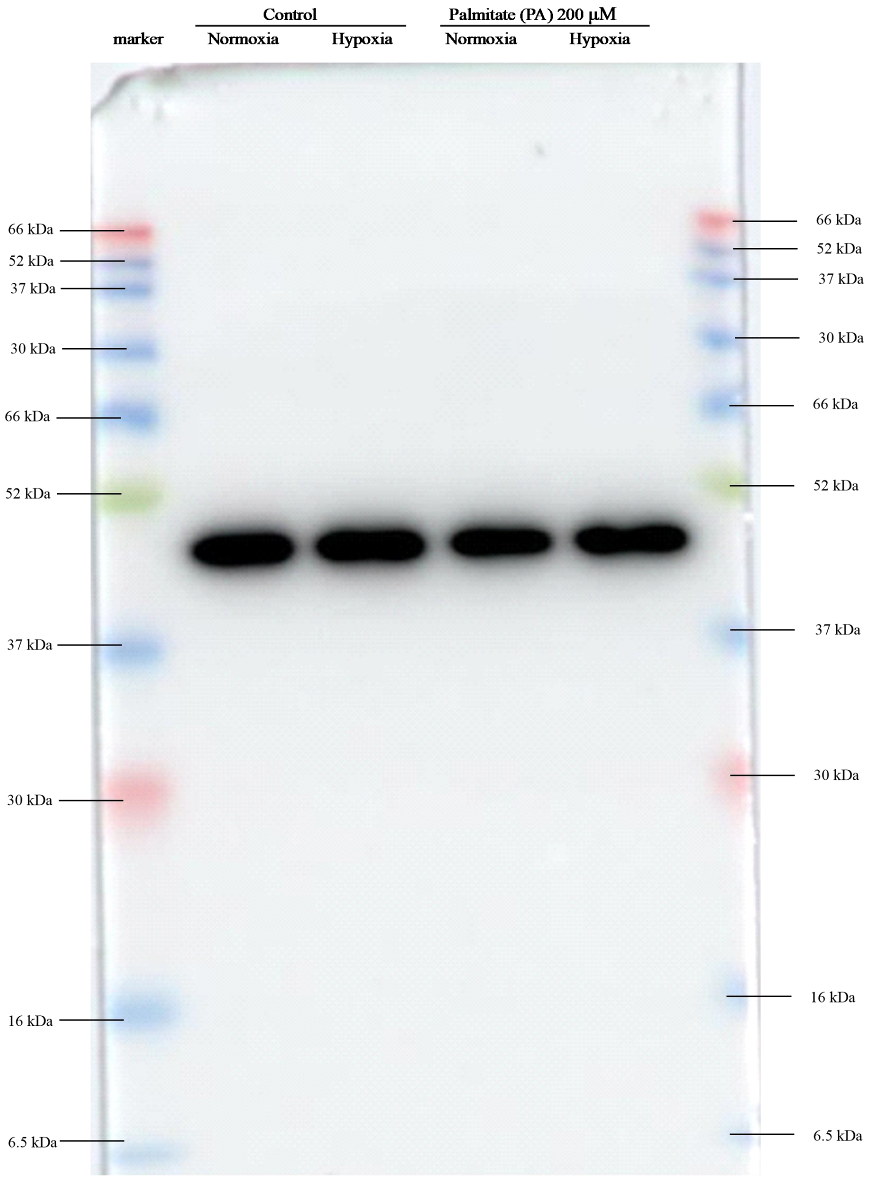

Total oxphosphate Rodent WB antibody Cocktails 20-55 kDa

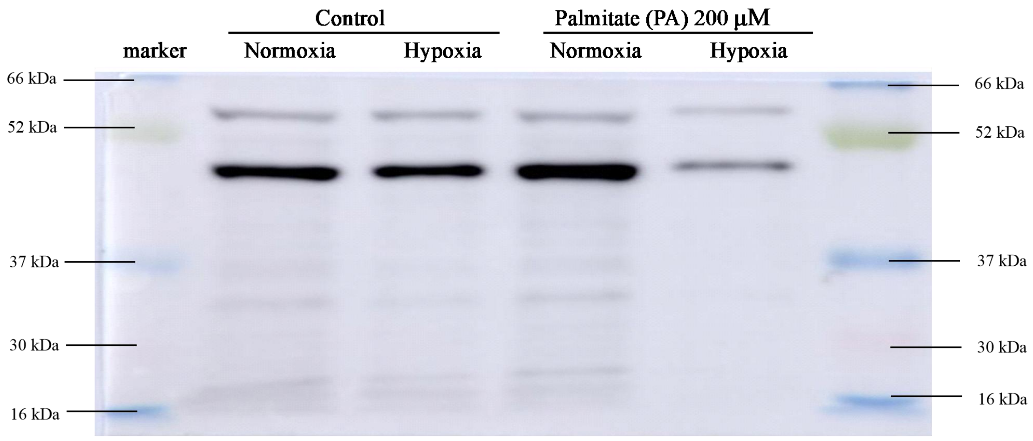

Anti-VDAC1/Porin Antibody 31kDa

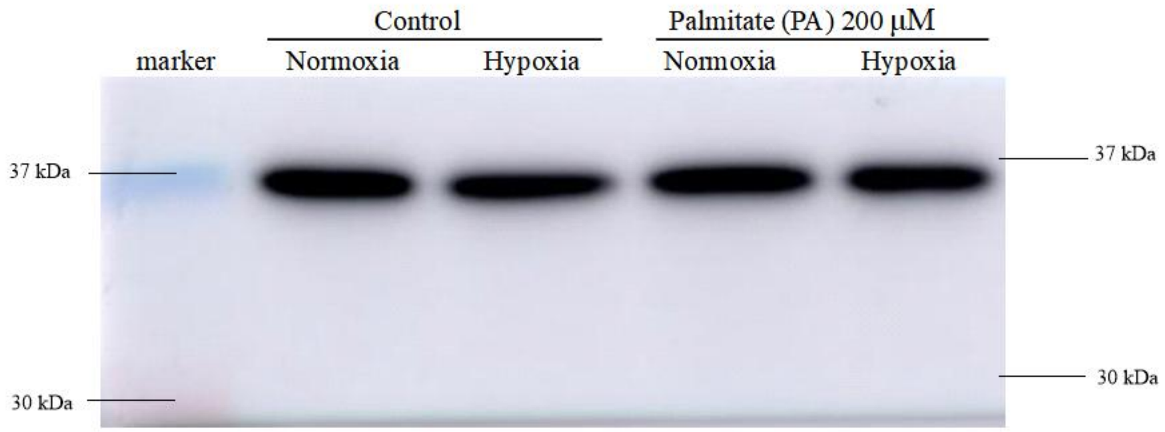

Fig. S7 Wounding Healing Assay and Western blot for glutamine deprived media to treatment the Lx-2 cells with and without absence of hypoxia.

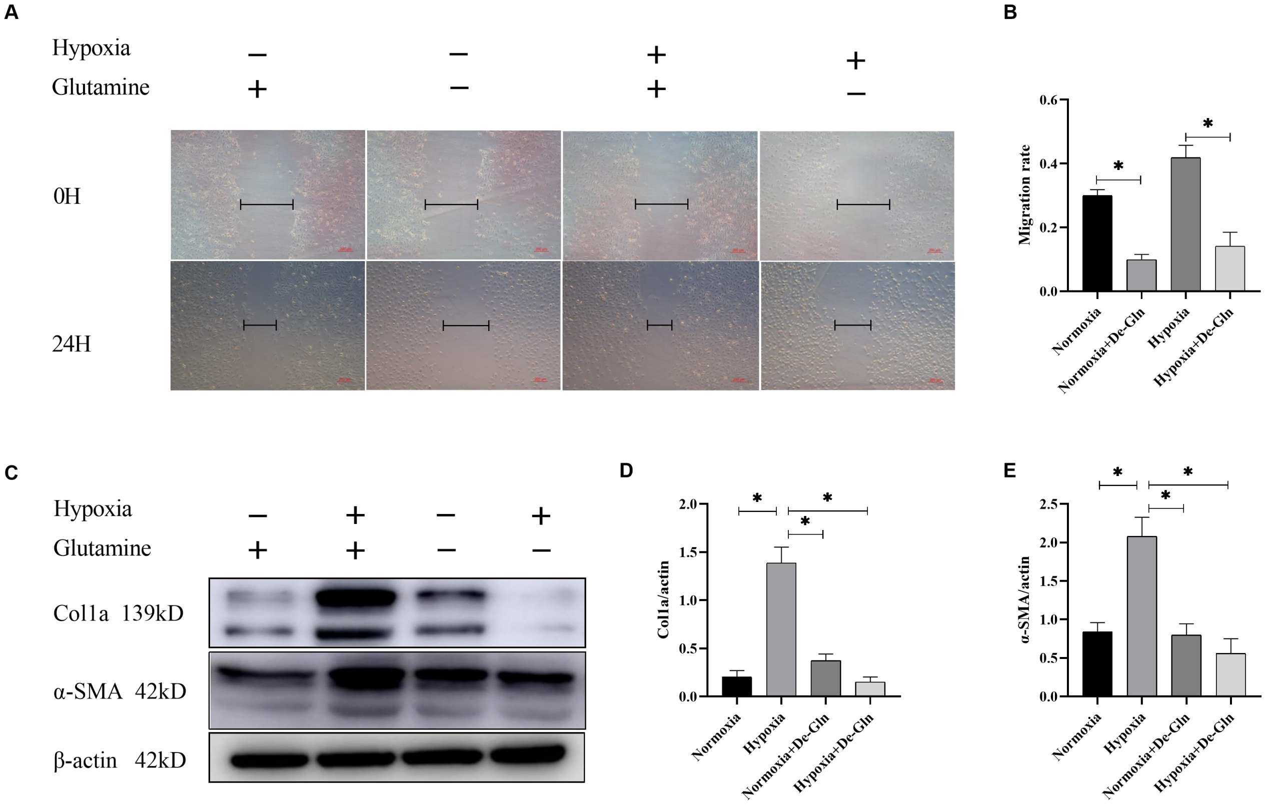

The percentage of wound closure was determined according to the following formula: wound closure rate = [wound area (0 h) – wound area (24 h)]/wound area (0 h) .

Fig. S8 Verification of interference effects for knock down from the picked and applied YAP shRNAs plasmids from the three biosynthesized candidate plasmids, which has been showned in figure 6 and figure 7

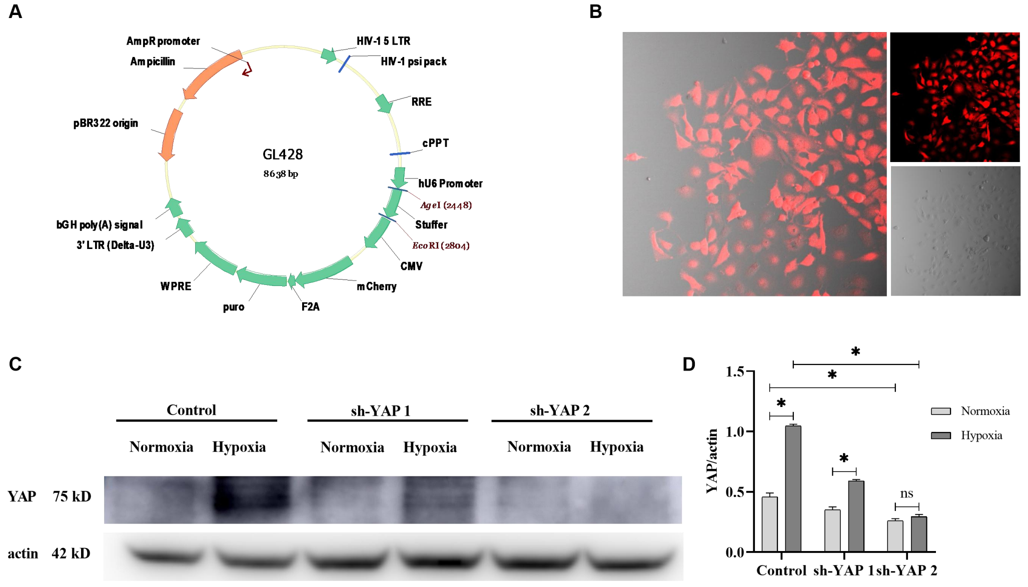

Synthesis and construction of shRNA interference fragments of Human YAP1 using lentivirus as carrier by Heyuan Biotechnology (Shanghai) Company Limited. The vector map of lentivirus interference vector pSLenti-U6-shRNA-CMV-mCherry-F2A-Puro-WPRE is as follows (Fig. S7 A). Constructing two sh-YAP cell models by introducing lentivirus carrying Human YAP1-shRNA interference fragments into LX-2 cells (Fig. S7 B). Protein extraction from cell models after hypoxia stimulation for western blot experiments to verify the effect of shRNA interference. The verification results are shown in the figure S7 C and D. The results indicate that sh-YAP 2 has the best interference effect, and the expression level of YAP protein is about 28.07% of the hypoxic control group, significantly reduced compared to the control group, with an absolute value decrease of 71.93%.

Fig. S9 Verification of interference effects from the picked and applied HIF-2 α shRNA plasmid from the biosynthesized candidate plasmids, which has been showed in figure 6 and figure 7

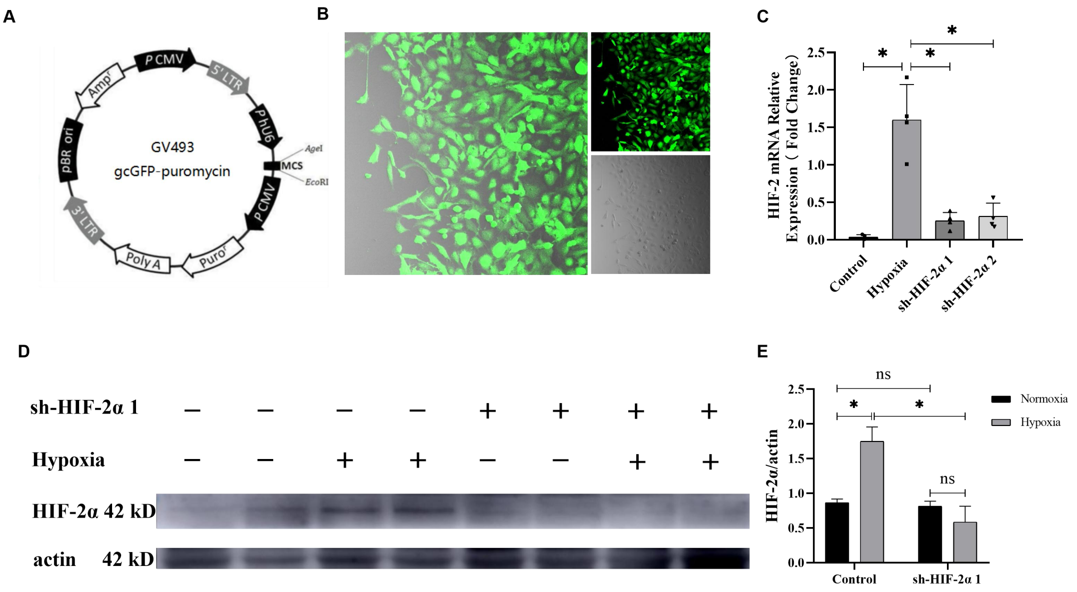

Shanghai Jikai Gene Technology Company Limited constructs shRNA interference fragments of Human HIF-2α using lentivirus as a vector.The slow virus interference vector is hU6-MCS-Ubiquitin-firefly_Luciferase-IRES-puromycin. The carrier map is as follows (Fig. S6 A). Two sh-HIF-2α cell models were constructed by introducing shRNA interference fragments containing Human HIF-2α into LX-2 cells via lentiviral vectors (Fig. S6 B). Extract total RNA after hypoxic stimulation for qRT-PCR validation of interference effect. LX-2 cells transfected with shRNA were subjected to hypoxia stimulation and total RNA was extracted for qRT PCR detection. The results showed a significant decrease in HIF-2α expression levels, with sh-HIF-2α 1 showing the best effect (Fig. S6 C). Select the interference model constructed by sh-HIF-2α 1 for hypoxia stimulation and extract proteins for protein blotting experiments to verify the interference effect. The results are shown in the figure S6 D. The results indicate that sh-HIF-2α 1 has the best interference effect, and the expression level of HIF-2α protein is about 33.43 % of the hypoxic control group, significantly reduced compared to the control group, with an absolute value decrease of 66.57 % (Fig. S6 E) .

Table S1 List of Amino acid metabolism analysis information table for enrolled volunteers

Index	NASH	NASH+CMS
Patients (n)	6	6
Age	40 (33.25, 51)	45.5(35.5,51)
BMI	26.47 (22.87, 30.44)	30.48 (25.71, 33.95)
Weight	165.5 (160, 173.5)	172 (165.75, 180)
Hemoglobin	215(213, 227.5)	218(214.5, 227.5)
Fat (area in %)	59 (46, 75)	74 (60,80)
Inflammation (0-3)	2 (1, 2)	2 (1, 2)
Fibrosis (0-4)	2(1,3)	3 (1, 3)
NAS (0-8)	5 (5,6)	6 (6, 7)

NASH

Table S2 List of all antibodies used in the immunofluorescence

antibody	Manufacturer	Cat. No	Source	Conjugation	Dilution
Anti-HIF-2-alpha antibody	abcom	ab109616	Rabbit	-	1:200
Anti-active YAP1 antibody	abcom	ab205270	Rabbit	-	1:500
α-Smooth Muscle Actin	CST	19245	Rabbit	-	1:400
GLS Recombinant antibody	Proteintech Group	81486-1-RR	Rabbit	-	1:500
HIF-1α	Servicebio	GB114936	Rabbit	-	1:1000
HIF-2α	Servicebio	GB11864	Rabbit	-	1:1000
α-SMA	Servicebio	GB111364	Rabbit	-	1:200
HRP-labelled goat anti-rabbit IgG	Servicebio	GB23303	Goat	HRP	1:200
Alexa Fluor 488-labelled goat anti-rabbit IgG	Servicebio	GB25303	Goat	Ex: 495nm, Em: 519nm	1:400
Alexa Fluor 647 Rabbit monoclonal to active YAP1 (Alexa Fluor® 647)	abcom	ab225440	Rabbit	Ex: 652nm, Em: 668nm	1:100
Goat Anti-Rabbit IgG H&L (Alexa Fluor® 488)	abcom	ab150077	Rabbit	Ex: 495nm, Em: 519nm	1:600

Table S3 List of all antibodies used in the western blot

antibody	Manufacturer	Cat. No	Source	Molecular weight(kDa)	Dilution
Anti-HIF-1 alpha antibody	abcom	ab179483	Rabbit	92	1:1000
Anti-active YAP1 antibody	abcom	ab205270	Rabbit	75	1:1000
Anti-YAP1 (phospho S127) antibody	abcom	ab76252	Rabbit	75	1:3000
Anti-Collagen I antibody	abcom	ab260043	Rabbit	139 ,220	1:1000
Total OXPHOS Rodent WB antibody Cocktail	abcom	ab110413	Mouse	20-55	1:1000
Anti-VDAC1/Porin	abcom	ab306581	Rabbit	31	1:1000
HIF-2Alpha （D6T8V) Rabit mAb	CST	59973S	Rabbit	120	1:1000
α-Smooth Muscle Actin	CST	19245	Rabbit	42	1:1000
α/β-Tubulin Antibody	CST	2148S	Rabbit	55,52	1:1000
Anti-β-Actin Antibody	sigma	A5441	Mouse	42	1:10000
anti-rabbit IgG HRP-linked antibody	CST	7074p2	goat	-	1:2000
anti-mouse IgG HRP-linked antibody	CST	7076P2	horse	-	1:2000
